# Immunometabolic interference between cancer and COVID-19

**DOI:** 10.3389/fimmu.2023.1168455

**Published:** 2023-03-29

**Authors:** Francesca Maria Consonni, Barbara Durante, Marcello Manfredi, Augusto Bleve, Chiara Pandolfo, Valentina Garlatti, Virginia Vita Vanella, Emilio Marengo, Elettra Barberis, Barbara Bottazzi, Sara Bombace, Ilaria My, Gianluigi Condorelli, Valter Torri, Antonio Sica

**Affiliations:** ^1^ Department of Pharmaceutical Sciences, University of Piemonte Orientale “A. Avogadro”, Novara, Italy; ^2^ IRCCS Humanitas Clinical and Research Centre, Rozzano, Milan, Italy; ^3^ Department of Translational Medicine, University of Piemonte Orientale, Novara, Italy; ^4^ Center for Translational Research on Autoimmune and Allergic Diseases, University of Piemonte Orientale, Novara, Italy; ^5^ Department of Sciences and Technological Innovation, University of Piemonte Orientale, Alessandria, Italy; ^6^ Department of Cardiovascular Medicine, Humanitas Clinical and Research Center, Rozzano, Milan, Italy; ^7^ Department of Biomedical Sciences, Humanitas University, Pieve Emanuele-Milan, Italy; ^8^ Istituto di Ricerche Farmacologiche Mario Negri-IRCCS, Milan, Italy

**Keywords:** COVID - 19, cancer, metabolism, immunity, inflammation

## Abstract

Even though cancer patients are generally considered more susceptible to severe acute respiratory syndrome coronavirus 2 (SARS-CoV-2) infection, the mechanisms driving their predisposition to severe forms of coronavirus disease 2019 (COVID-19) have not yet been deciphered. Since metabolic disorders are associated with homeostatic frailty, which increases the risk of infection and cancer, we asked whether we could identify immunometabolic pathways intersecting with cancer and SARS-CoV-2 infection. Thanks to a combined flow cytometry and multiomics approach, here we show that the immunometabolic traits of COVID-19 cancer patients encompass alterations in the frequency and activation status of circulating myeloid and lymphoid subsets, and that these changes are associated with i) depletion of tryptophan and its related neuromediator tryptamine, ii) accumulation of immunosuppressive tryptophan metabolites (i.e., kynurenines), and iii) low nicotinamide adenine dinucleotide (NAD^+^) availability. This metabolic imbalance is accompanied by altered expression of inflammatory cytokines in peripheral blood mononuclear cells (PBMCs), with a distinctive downregulation of IL-6 and upregulation of IFNγ mRNA expression levels. Altogether, our findings indicate that cancer not only attenuates the inflammatory state in COVID-19 patients but also contributes to weakening their precarious metabolic state by interfering with NAD^+^-dependent immune homeostasis.

## Introduction

The pathophysiological changes and metabolic disorders leading to the subclinical phase of “homeostatic frailty” are common among the elderly and are associated with greater susceptibility to infection (1) and cancer (2). These changes lead to immune-senescence and imbalance between activation and resolution mechanisms of inflammation, resulting in high levels of inflammatory mediators, such as interleukin-6 (IL-6), tumor necrosis factor-α (TNF-α), and C-reactive protein (CRP) (3). Failure to keep in check inflammation is then thought to compromise several other metabolic and immune pathways (1). Intriguingly, severe COVID-19 patients display massive inflammation, possibly resulting from the loss of homeostatic robustness, which is clinically mitigated by the treatment with monoclonal antibodies against IL-6 (e.g., tocilizumab) (4) and/or corticosteroids (5).

In addition to aging, a number of other comorbidities (e.g., cancer, diabetes, hypertension, and cardiovascular disease), all with underlying chronic inflammatory and metabolic disorders, are associated with increased severity of COVID-19 (6). However, the net immunometabolic contribution of such comorbidities to severe COVID-19 is still poorly understood. What we do know is that cancer and COVID-19 exploit distinct patterns of macrophage activation, which promotes disease progression in their most severe forms (6).

Cancer progression is known to promote immunosuppression, often associated with the production of anti-inflammatory and immunosuppressive cytokines (e.g., IL-10, and TGFβ) by alternative/M2 activated myeloid cells, especially macrophages (7, 8). While an M2 activation state of macrophages is generally associated with cancer progression, the most severe forms of COVID-19 correlate with the development of macrophage activation syndrome (MAS), a state of systemic hyperinflammation characterized by a storm of M1-related cytokines (6). Intriguingly, the opposing M1 (pro-inflammatory) and M2 (anti-inflammatory) polarization states (9) are respectively driven by Th1- (i.e., interferon-γ) and Th2-polarizing cytokines (i.e., IL-4) (7), whose imbalance has been linked to a higher risk of COVID-19 mortality (10). Thus, given that M1 and M2 polarization are metabolically distinct states (11, 12), we asked whether the immunosuppressive and anti-inflammatory action of growing tumors would interfere with the immunometabolic profile induced by SARS-CoV-2 infection. We emphasize that our study refers to biological samples collected from COVID-19 patients hospitalized at the Humanitas hospital (Rozzano, Milan) in the period February 2020-October 2020, when the regions of northern Italy (i.e. Lombardy) showed a high prevalence of B.1 lineage (clade 20A) (13, 14).

## Methods

### Study participants

This study was approved by the Humanitas Clinical and Research Center Ethics Committee (study number 2490; identification 1366); the requirement for informed consent was waived.

Non-small cell lung cancer (NSCLC) patients at different stages of tumor progression were enrolled in the study after signing the Cancer Research Center Humanitas IRB-approved informed consent. All subjects were sex and aged mixed. The study was approved by the Italian Ministry of Health (Nos. 97/2014-PR and 25/2018-PR).

The COVID-19 patients recruited for this study were hospitalized at the Humanitas Research Hospital (ICH) and had a nasopharyngeal swab RT-PCR-confirmed SARS-CoV-2 infection. Upon hospital admission, COVID-19 patients underwent complete laboratory testing (**Tables 1**, **S1**). These patients were classified according to the presence or absence of cancer as comorbidity. Other clinical characteristics are listed in **Table 1**. Consecutive patients admitted to our COVID-19 department were enrolled in the study and the following clinical information were collected: age, sex, respiratory support, pharmacological treatment, cardiovascular risk factor profile, serum biomarkers (IL-6, CRP, D-dimer) and a diagnosis of active cancer on admission. In total we recruited 50 COVID-19 patients (COV), 25 COVID-19 patients with cancer (COV/CA), 15 NSCLC cancer patients (CA) and 14 healthy donors (HD). In detail, for flow cytometry analysis we used 14 HD, 15 CA, 21 COV and 8 COV/CA patients, whereas for the lipidomic, metabolomic and proteomic analysis a total number of 11 CA, 23 COV and 13 COV/CA were analyzed.

**Table 1:** Demographics and clinical characteristics of patients infected with SARS-CoV-2, with and without oncological disease, involved in the study.

Characteristics	COVID No Cancer	COVID Cancer	P Value Univ.	Test	Pvalue Multiv.
**Male N(%)**	35 (70%)	18 (72%)	0.859	^	
**Age mean +/- SD**	71.5 +/- 14.3	67.0+/- 12.3	0.184	*	
**Obesity N(%)**	10 (20%)	2 (8%)	0.184	^	
**Smoke N(%)**	3 (6%)	0 (0%)	0.214	^	
**Dyslipidemia N(%)**	3 (6%)	0 (0%)	0.214	^	
**Hypertension N(%)**	32 (64%)	5 (20%)	<0.001	^	<0.001
**Diabetes Mellitus N(%)**	22 (44%)	5 (20%)	0.043	^	
**Vasculopathy N(%)**	10 (20%)	2 (8%)	0.184	^	0.091
**COPD N(%)**	6 (12%)	2 (12%)	1.000	^	
**Coronary artery disease N (%)**	10 (20%)	4 (16%)	0.677	^	
**Il6 median IQR**	22.5 (15-67)	28 (12-64)	0.937	**	
**CPR median IQR**	6 (3-13)	5 (1.95-7.19)	0.150	**	
**D dimer median IQR**	607 (313-1461)	532 (306-1087)	0.320	**	0.074
**Tocilizumab**	1 (2%)	0 (0%)	0.479	^	
**Steroids**	21 (42%)	7 (28%)	0.240	^	
**Death**	8 (16%)	4 (16%)	1.000	^	
**Respiratory support**			0.010	^^	
** None**	8 (16%)	9 (36%)			
** O2**	20 (40%)	10 (40%)			
** Non invasive**	11 (22%)	6 (24%)			
** Invasive**	11 (22%)	0 (0%)			

^= χ^2^ test; ^^= χ ^2^ test for trend; *Student T test; ** Wilcoxon test.

Respiratory support: patients with Non-invasive ventilator and full ventilator were considered severe.

### Plasma and PBMCs isolation

Peripheral blood mononuclear cells (PBMCs) and plasma were isolated from whole blood samples collected in EDTA vacutainer tubes. All blood samples were analyzed within 3 h from the collection time. Briefly, whole blood samples were spun down at 800 x g for 10 min, blood plasma was collected and stored at -80°C until analysis, PBMCs were isolated with Limpholyte Cell Separation Media (Euroclone), and purified cells were collected and preserved in 10% DMSO/FBS at -80°C until further use. After cell count, 10^6^ cells were lysed in TRIzol and store at -80°C before used for qPCR analysis.

### Statistics

Demographics and clinical characteristics of patients infected with SARS-CoV-2, with and without oncological disease, involved in the study were summarized by means of mean median quartiles and extreme values for continuous data, and with absolute and relative frequencies for categorical and ordinal data. Data were reported for the overall sample population and by presence/absence of cancer. Comparison of variables distributions between cancer and non-cancer patients was tested with parametric and non-parametric approaches. A multivariable logistic model was then used to identify variables independently associated with cancer; a backward selection procedure (with cut-off level to remain in the model =0.10), was used for variable selection.

### PBMCs immunophenotyping

Cells were thawed in complete RPMI medium and then washed in 1X PBS; 1.5 x 10^6^ cells were resuspended in 50 µl of staining buffer [PBS, 2% FBS, 2 mM ethylenediaminetetraacetic acid (EDTA)] containing Aqua LIVE/ead-405-nm staining (1:800, Biolegend Cat. No. 423102) and the indicated antibody directed against cell surface or intracellular markers. Cells were incubated with the following anti-human antibodies for 20 min at 4°C: CD33 PerCP Cy5.5 (WM53) (0.6:100, Biolegend cat No. 303402), CD14 BV650 (M5E2) (1.25:100, Biolegend cat. No. 301835), CD16 BV711 (3G8) (0.3:100, Biolegend cat. No. 302044), HLA-DR BV421 (L243) (1.25:100, Biolegend Cat No. 307636), CD15 BV786 (HI98) (1.25:100, BD Biosciences cat. No. 563838), CD11c BUV661 (B-ly6) (0.6:100, BD Biosciences cat. No. 612968), CD123 BUV395 (7G3) (0.6:100, BD Biosciences cat. No. 564195), CD38 PE-Cy7 (HIT2) (1.25:100, Invitrogen cat. No. 25-0389-42), CD39 PE (MZ18-23C8) (0.6:100, MACS Miltenyi Biotec Inc. cat. No.130-118-668), CD39 FITC (MZ18-23C8) (1.25:100, MACS Miltenyi Biotec Inc. cat. No. 130-125-113), CD3 BV650 (OKT3) (1.25:100, Biolegend cat. No.317324), CD4 BV570 (OKT4) (2.5:100, Biolegend cat. No. 317445), CD8 BV786 (RPA-T8) (0.6:100, BD Biosciences cat. No. 557085), CD19 AF-700 (HIB19) (2.5:100, BD Biosciences cat. No. 561031), CD45RA APC-Cy7 (HI100) (1.25:100, BD Biosciences cat. No. 560674), CD45RO BUV395 (UCHL1) (1.25:100, BD Biosciences 564292), CD27 PE-Cy5 (O323) (1.25:100, Invitrogen cat. No. 15295964), CD16 BV605 (3G8) (0.6:100, Biolegend 302040), CD3 BUV496 (UCHT1) (1.25:100, BD Biosciences cat. No. 612940), CD66b PE-Cy7(G10F5) (0.6:100 Biolegend cat. No. 305116) and IgD FITC (IA6-2) (1.25:100, Invitrogen cat. No. 11-9868-42).

Upon cell permeabilization with Foxp3/Transcription Factor Staining Buffer Set (eBioscience), cells were stained for 20 min at 4°C with PARP1 PE (HLNC4) (1.25:100, Invitrogen cat. No. 12-6668-42) and with unconjugated mouse anti-human NAMPT (OMNI 379) (1:100, Adipogen cat. No. AG-20A-0034), unconjugated rabbit anti-human pSIRT (Ser47) (1:100, Invitrogen cat. No. PA5-17391), goat anti-rabbit Alexa Fluor 647-conjugated antibody (1:500, ThermoFisher cat. No. A27040), goat anti-mouse Alexa Fluor 647-conjugated antibody (1:500, ThermoFisher cat. No. A20990). Samples were recorded using BD FACSymphony and analyzed with BD FACSDiva and FlowJo (9.3.2, 9.9.6 or 10.7.1) software. Results are expressed as MFI (mean fluorescent intensity) or as percentage of the gated cells among CD45^+^ cells.

### Quantitative PCR (qPCR)

Total RNA was extracted with TRIzol reagents (Invitrogen) according to the manufacturer’s instructions. Complementary DNA was synthesized by reverse transcription using High Capacity cDNA Archive Kit (Applied Biosystems, cat. No. 4368814). Quantitative real-time PCR was performed using SYBR Green PCR Master Mix (Applied Biosystems, cat. No. 4309155). All samples were analyzed using ViiA 7 Real-Time PCR system QuantStudio Real-Time PCR Software v1.6.1 (Applied Biosystems). Samples were normalized to *β-actin* expression and results were expressed as Δ Ct. Primer sequences: *TNFα* (F: ACGAACATCCAACCTTCCCA; R: CCCAATTCTCTTTTTGAGCCA), *IL-1β* (F: CCTACTCACTTAAAGCCCGCC; R: TTAGAACCAAATGTGGCCGTG), *IL-6* (F: AGAACAGATTTGAGAGTAGTGAGGAAC; R: GGCATTTGTGGTTGGGTCAGG), *CXCL10* (F: GGAAGCACTGCATCGATTTTG; R: CAGAATCGAAGGCCATCAAGA), *IL-10* (F: TTAAGGGTTACCTGGGTTGCCAAGC; R: TCTTGGTTCTCAGCTTGGGGCATCA), *IFN-β* (F: GACATCCCTGAGGAGATTAAGCA; R: GGAGCATCTCATAGATGGTCAATG), *IFNα1* (F: CCCACAGCCTGGATAACAG; R: ACTGGTTGCCATCAAACTCC), *IFNα2* (F: GACCTGGAAGCCTGTGTGAT, R: CAGGCACAAGGGCTGTATTT), *TGF-β* (F: ACTATTGCTTCAGCTCCACGGA, R: AAGTTGGCATGGTAGCCCCTTG), IFNγ (F: TTTGGGTTCTCTTGGGTGTTACT; R: CCTTTTTCGCTTCCCTCGTTTT), *β-ACTIN* (F:CCCAAGGCCAACCGCGAGAAGAT, R: GTCCCGGCCAGCCAGGTCCAG).

### Lipidomic analysis

PBMCs (1x10^6^) were extracted using a 1 mL solution of 75:25 IPA/H2O, after the addition of deuterated lipid standard (Splash Lipidomix^®^). The samples were vortexed and sonicated for 2 min and then incubated for 30 min at 4°C, under gentle, constant shaking. To remove debris and other impurities, the samples were centrifuged for 10 min at 3500*g* at 4°C. Subsequently, 1 mL of supernatant was collected and dried using a SpeedVac. The dried samples were reconstituted in 100 μL of MeOH containing the internal standard CUDA (12.5 ng/mL). The reconstituted lipids were analyzed by UHPLC Vanquish system (Thermo Scientific, Rodano, Italy) coupled with Orbitrap Q-Exactive Plus (Thermo Scientific). A reverse phase column was used for lipid separation (Hypersil Gold™ 150 × 2.1 mm, particle size 1.9 μm); the column was maintained at 45°C at a flow rate of 0.260 mL/min. Mobile phases and mass spectrometry parameters were the same as those reported by Masini *et. al.*, 2022 (15). The acquired raw data from the untargeted analysis were processed using MSDIAL software (Yokohama City, Kanagawa, Japan), version 4.24. This included the detection of peaks, MS2 data deconvolution, compound identification and the alignment of peaks across all samples. In order to obtain an estimated concentration expressed in μg/mL, the normalized areas were multiplied by the concentration of the internal standard. An in-house library of standards was also used for lipid identification. Statistical analysis was performed using MetaboAnalyst 5.0 software (www.metaboanalyst.org) and GraphPad Prism v. 8.

### Metabolomic analysis

Metabolomic analyses of plasma samples were conducted as previously reported (16). Briefly, plasma metabolites were obtained through protein precipitation with cold acetonitrile/isopropanol/water, followed by derivatization with methoxamine and BSTFA. The following internal standards were spiked in each sample and were used for data normalization and instrument stability monitoring: tridecanoic acid (0.5 mg/mL), palmitic acid d31 (0.5 mg/mL), stearic acid d35(0.5 mg/mL), glycine d4 (10.07 mg/mL) and hexadecane (1.0 mg/mL). Quality control samples (QC) containing spiked standards were also acquired every 10 samples. Small molecules were analyzed by LECO Pegasus BT 4D GCXGC/TOFMS (Leco Corp., St. Josef, MI, USA) equipped with LECO dual stage quad jet thermal modulator. The GC part of the instrument was an Agilent 7890 gas chromatograph (Agilent Technologies, Palo Alto, CA, USA) equipped with a split/splitless injector. The first-dimension column was a 30 m Rxi-5Sil (Restek Corp., Bellefonte, PA, USA) MS capillary column with an internal diameter of 0.25 mm and a stationary phase film thickness of 0.25 μm. The second-dimension chromatographic column was a 2 m Rxi-17Sil MS (Restek Corp., Bellefonte, PA, USA) with a diameter of 0.25 mm and a film thickness of 0.25 μm. High-purity helium (99.9999%) was used as carrier gas, with a flow rate of 1.4 mL/min. One μL of sample was injected in splitless mode at 250°C. The temperature program consisted of an initial temperature of 100°C for 2 min, which was then increased to 330°C at a rate of 20°C/min up and maintained at this temperature for 2 min. The secondary column was maintained at +5°C relative to the GC oven temperature of the first column. The programming rate was the same as that used for the two columns. Electron impact ionization was applied (70 eV). The ion source temperature was set at 250°C, while the mass range was 25 to 550 m/z, with an extraction frequency of 32 kHz. The acquisition rates were 200 spectra/s. The modulation period for the bi-dimensional analysis was 4 s for the entire run. The modulator temperature offset was set at +15°C relative to the secondary oven temperature, while the transfer line was set at 280°C.

The chromatograms were acquired in total ion current mode. Peaks with signal-to-noise (S/N) value lower than 500.0 were discarded. The raw data were processed with ChromaTOF version 5.31. Mass spectral assignment was performed by matching data with NIST MS Search 2.3 libraries and FiehnLib. An in-house library of standards was also used for small molecule identification. MetaboAnalyst 5.0 software (www.metaboanalyst.org) was used for statistical analysis.

### Proteomic analysis

PBMCs (1x10^6^) of were lysed in 200 μL of RIPA buffer (50mM Tris HCl pH 7.2, 0.05%SDS) by sonication. Cold acetone was used for protein precipitation followed by resuspension in 100 mM NH_4_HCO_3_. Protein content was measured by Bradford Protein Assay (Sigma-Aldrich, St. Louis, MO). Proteins (50 µg) were subjected to reduction with DTT, alkylation with iodoacetamide and tryptic digestion at 37 °C overnight. Peptides were then desalted with Discovery^®^ DSC-18 solid phase extraction (SPE) 96-well plate (25 mg/well) (Sigma-Aldrich Inc., St. Louis, MO, USA) and then analyzed through label-free LC–MS/MS, performed by using a micro-LC system (Eksigent Technologies, Dublin, USA) interfaced with a 5600+ TripleTOF mass spectrometer (Sciex, Concord, Canada). Peptides were separated using a Halo C18 column (0.5×100 mm, 2.7 μm; Eksigent Technologies Dublin, USA). The reverse phase LC solvents included solvent A (99.9% water +0.1% formic acid) and solvent B (99.9% acetonitrile +0.1% formic acid). The separation was performed by using a 30-min gradient at a flow rate of 15 μL/min, with an increasing concentration of solvent B from 2% to 40%. For identification purposes, experiments were set to obtain a high-resolution TOF-MS scan over a mass range of 100–1500 m/z, followed by an MS/MS product ion scan from 200 to 1250 Da (accumulation time of 5.0 ms), with the abundance threshold set at 30 cps (35 candidate ions can be monitored during every cycle). The ion source parameters in electrospray positive mode were set as follows: curtain gas (N2) at 25 psig, nebulizer gas GAS1 at 25 psig, GAS2 at 20 psig, ion spray voltage floating (ISVF) at 5000 V, source temperature at 450°C and declustering potential at 25 V. Using the same conditions as described above, a SWATH acquisition using DIA was carried out for the label-free quantification process using an accumulation time of 40 ms per 25-Da swath (36 swaths in total). The MS data were acquired with Analyst TF 1.7 (AB SCIEX, Concord, Canada) (17).

The mass spectrometry files were searched against the Swiss-Prot human database (42,271 sequences) by means of Protein Pilot software v. 4.2 (SCIEX, Concord, Canada) using the following parameters: cysteine alkylation, digestion by trypsin, no special factors and false discovery rate (FDR) at 1%. We also employed the Mascot v. 2.4 (Matrix Science Inc., Boston, USA) according to the following parameters: trypsin digestion (two missed cleavages), ESI-QUAD-TOF setting, carbamidomethyl cysteine as fixed modification and oxidized methionine as variable modification. An assay tolerance of 50 ppm was specified for peptide mass tolerance, and 0.1 Da for MS/MS tolerance. A target-decoy database search was performed, and FDR was fixed at 1%. The quantification was carried out with PeakView 2.2 and MarkerView 1.2. (Sciex, Concord, ON, Canada) according to the following parameters: six peptides per protein and six transitions per peptide. Statistical analysis and related graphical representations were done using GraphPad Prism v. 8 and MetaboAnalyst software (www.metaboanalyst.org). Ingenuity Pathways Analysis (IPA) software (Qiagen, Redwood City, CA, USA) and FunRich (http://www.funrich.org) were used for bioinformatics analysis.

The mass spectrometry data have been deposited to the ProteomeXchange Consortium *via* the PRIDE partner repository with the dataset identifier PXD040683.

### Intracellular NAD levels quantification

PBMCs (50.000 cells) purified from HD, CA, COV and COV/CA patients were cultured in completed RPMI medium (10% fetal bovine serum (FBS), 2 mM L-glutamine and 100 U/ml Penicillin and 100 µg/ml Streptomycin), supplemented with 800 µM Nicotinamide (Duchefa Biochemie) or 100 µM Apigenin (Merk) or 500 µM Leucine (Merk) or cell culture media for 1 h. Intracellular NAD+ levels were then quantified *via* NAD/NADH-Glo Assays (Promega). Luminescence was quantified with microplate reader Synergy 2 (Bio-Tek instrument INC).

## Results

### Alterations of circulating immune cells in cancer patients with or without COVID-19

As COVID-19 severity correlates with hyperactivation of the immune system, typified by MAS, lymphopenia, endothelial dysfunctions, and immunothrombosis (18), we first assessed the expression of biomarkers of COVID-19 severity, such as IL-6, CRP, and D-dimer (1, 19, 20), in the plasma from COVID-19-only (COV) (n=50) vs COVID-19 cancer (COV/CA) patients (n=25) (**Table 1**; **Supplemental Table 4**). Besides confirming the elevation of these markers in the COV group, we found that COV/CA patients consistently displayed decreased plasma levels of CRP and D-dimer (**Table 1**). Furthermore, multivariate logistic analysis, adopting a backward conditional procedure at a cut-off level of 0.10, revealed that hypertension, vasculopathy, and D-dimer formation were inversely associated with cancer occurrence (hypertension OR = 0.09; 95%CI (0.02-0.31); vasculopathy OR = 0.21; 95%CI (0.04-1.28); D-dimer OR x 100 units increase = 0.94 95%CI (0.89-1.01).

**Figure 1:**
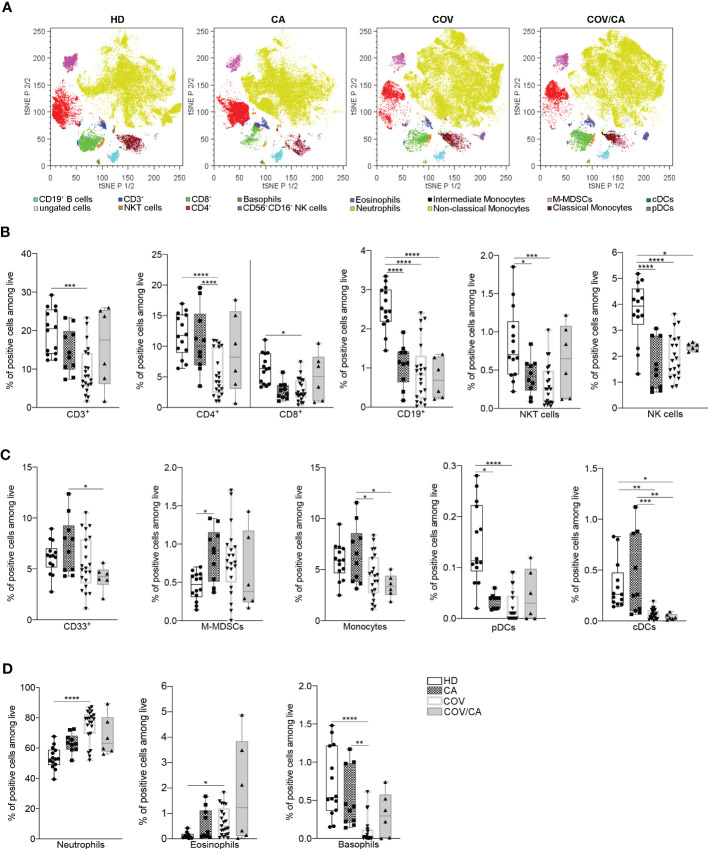
Influence of cancer and COVID-19 on the frequencies of circulating leukocyte subsets. **(A)** t-SNE analysis of circulating immune cells from 44 samples [10 healthy donors (HD); 6 cancer patients (CA); 22 COVID-19 patients (COV); and 6 COVID-19 and cancer patients (COV/CA)] gated on CD45^+^ cells after exclusion of dead cells. The dot plots show the distribution of the indicated immune cell population. **(B–D)** Box-and-whisker plots representing frequencies of **(B)** CD3^+^, CD4^+^ and CD8^+^ T cells, CD19^+^ B cells, NKT and CD56^+^CD16^+^ NK cells, **(C)** CD33^+^ myeloid cells, M-MDSCs, monocytes, pDCs and DCs, **(D)** neutrophils, eosinophils and basophils. Fresh blood samples were collected from HD (*n* = 10 or 14), CA (*n* = 10), COV (*n* = 21) and COV/CA (*n* = 6) patients. Boxplot representation (centre line, mean; box limits, upper and lower quartiles; whiskers, range; points, data points per patient). Statistical significance of differences between patient groups was calculated by two-way ANOVA with Sidak’s multiple comparisons test or one-way ANOVA followed by Tukey’s multiple comparison test or a Kruskal-Wallis test followed by Dunn′s multiple comparisons test. *P < 0.05, **P < 0.01, ***P < 0.001, ****P < 0.0001 between selected relevant comparisons.

Based on these results, we asked whether cancer would affect the immune compartment in COVID-19 patients. To determine the frequencies of the main circulating immune cell subsets [i.e., CD3^+^, CD4^+^, CD8^+^, B lymphocytes, neutrophils, eosinophils, basophils, monocytes, classical and plasmacytoid dendritic cells (cDCs and pDCs), NK and NKT cells], whole blood samples were collected within 2 days of hospital admission and analyzed by means of a 14-multicolor flow cytometry panel. t-distributed stochastic neighbor embedding (t-SNE) analysis of myeloid and lymphoid cell subsets detected significant differences among the four cohorts enrolled in the study [i.e., healthy donors (HD), CA, COV, and COV/CA] (**Figures 1A–D**; **Supplemetal Figure 6A**). All patients, regardless of the group they belonged to, differed from each other in their immune cell frequency and distribution (**Figure 1A**). In good agreement with previous reports (21, 22), in COV vs HD we found a remarkable drop in the percentage of CD3^+^, CD4^+^ and CD8^+^ T cells (**Figure 1B**), monocytes, cDCs (**Figure 1C**) and basophils (**Figure 1D**). These immune cell subsets showed a similar distribution pattern in CA patients, albeit to different extents (**Figures 1B–D**). The number of CD19^+^ B cells, NKT, NK and pDCs was also significantly decreased in COV vs HD (**Figures 1B, C**). Moreover, in COV/CA vs COV we observed a slight increase in the percentage of NKT, NK, CD4^+^ and CD8^+^ T and B cells (**Figure 1B**). Given that cDCs and pDCs play a role in the production of antiviral type I interferons (23), their low number in COV and COV/CA with respect to HD and CA alone (**Figure 1C**) is suggestive of a limited activation of antiviral T and NK cell functions in SARS-CoV-2-infected patients (24). Moreover, as observed for the lymphoid subsets (**Figure 1B**), and supporting a possible contrasting effect of cancer on the inflammatory response elicited by SARS-CoV-2 infection, the increased number of neutrophils and the decreased basophil count, both previously linked to worsening conditions in COVID-19 patients (25–27), were attenuated in COV/CA patients (**Figure 1D**).

### Activation state of circulating immune cell subsets in COVID-19 vs COVID-19 cancer patients

**Figure 2:**
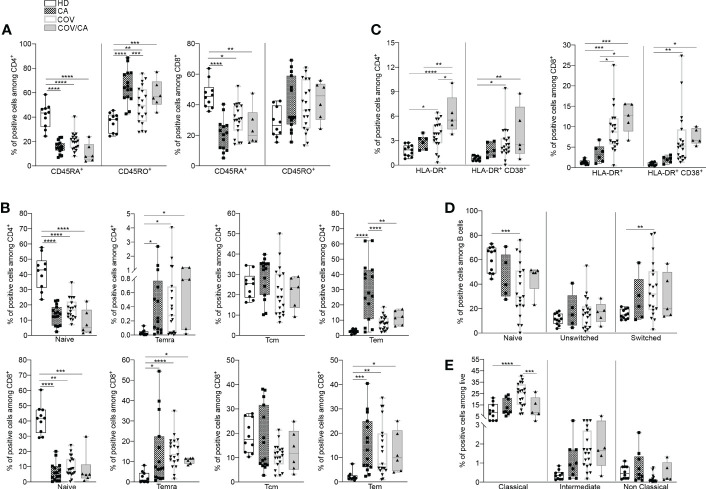
Cancer and COVID-19 affect the activation state of circulating immune cell subsets. **(A)** Box-and-whisker plots representing frequencies of CD45RA^+^ and CD45RO^+^ cells in CD4^+^ (left) and CD8^+^ (right) populations in freshly isolated PBMCs from HD, CA, COV and COV/CA patients (HD (*n* = 10), CA (*n* = 15), COV (*n* = 19) and COV/CA (*n* = 6)). **(B, C)** Box-and-whisker plots representing proportions of indicated lymphoid cell subsets (i.e., naïve, Temra, Tcm, Tem) in CD4^+^ (B, top) and CD8^+^ (**B**, bottom) populations, (HD (*n* = 10), CA (*n* = 15), COV (*n* = 19) and COV/CA (*n* = 6), and of HLA-DR^+^ and CD38^+^/HLA-DR CD4^+^ (**C**, left) and CD8^+^ (**C**, right) T cells in freshly isolated PBMCs as above (HD (*n* = 10), CA (*n* = 6), COV (*n* = 19) and COV/CA (*n* = 5)). **(D, E)** Box-and-whisker plots representing frequencies of indicated **(D)** B cell subsets (naïve, switched, and unswitched) (HD (*n* = 11), CA (*n* = 5), COV (*n* = 19) and COV/CA (*n* = 5)) and **(E)** of monocytes subsets (classical, intermediate and non-classical) in freshly isolated PBMCs as above (HD (*n* = 10), CA (*n* = 9), COV (*n* = 19) and COV/CA (*n* = 5)). Boxplot representation (centre line, mean; box limits, upper and lower quartiles; whiskers, range; points, data points per patient). The statistical significance of differences between patient groups was calculated using two-way ANOVA with Sidak’s multiple comparisons test or one-way ANOVA followed by Tukey’s multiple comparison test or a Kruskal-Wallis test followed by Dunn′s multiple comparisons test. *P < 0.05, **P < 0.01, ***P < 0.001, ****P < 0.0001 between selected relevant comparisons.

We next determined the functional state of both myeloid and lymphoid subsets in the different patient cohorts (**Supplemental Figures 6B, C**). In comparison with HD, CA, COV and COV/CA patients displayed reduced CD45RA^+^ cells, in both CD4^+^ and CD8^+^ populations, paralleled by accumulation of their memory CD45RO^+^ counterparts (**Figure 2A**). Since CD45RA^+^ T cells include both naïve and terminally differentiated effector cells (Temra), we used CD45RA, CD45RO, CD27 and CCR7 surface markers to detect naïve (CD45RA^+^CD45RO^-^CCR7^+^CD27^+^), Temra (CD45RA^+^CD45RO^-^CCR7^-^CD27^-^), as well as central (Tcm, CD45RA^-^CD45RO^+^CCR7^+^CD27^+^) and effector (Tem, CD45RA^-^CD45RO^+^CC R7^-^CD27^-^) memory T cells. Consistent with a state of immune diversion, in CA, COV and COV/CA we observed a significant drop in the frequency of naïve T cells (**Figure 2A**), whereas the percentages of Tem and Temra subsets were both increased (**Figure 2B**). In contrast, Tcm frequency was unchanged (**Figure 2B**). Lastly, the increased frequency of activated HLA-DR^+^ and CD38^+^/HLA-DR^+^ T cells observed in COV vs HD was further exacerbated in COV/CA patients (**Figure 2C**). Collectively, these data support a model in which SARS-CoV-2 infection promotes aberrant T lymphocyte overactivation and terminal differentiation. Indeed, while CD38 is selectively expressed during activation of a subset of mature T cells, characterized by reduced proliferation and enhanced cytokine production (28), CD38+HLA-DR+ CD8+ T cells are known to play contradictory roles in SARS-CoV-2 infection (29). In a similar trend, we observed that blood B cell differentiation in VOC patients is directed towards a more mature phenotype (i.e., CD27^+^IgD^-^) (**Figure 2D**). Overall, these observations highlight quantitative and qualitative alterations of the lymphoid repertoire in COVID-19 patients and define the relative contribution of cancer comorbidity to this phenomenon.

Since circulating monocytes are the most proximal precursors of macrophages, and the latter are accredited players in the severity of both COVID-19 (6, 30) and oncological diseases (7, 31), we next sought to determine the distribution of monocyte subsets in our study cohorts. A shown in **Figure 2E**, we observed a significant redistribution of these subsets (i.e., classical, intermediate and non-classical) (32) in CA, COV and COV/CA vs HD. Whereas classical monocytes have a pro-inflammatory phenotype (32), non-classical monocytes are patrolling phagocytosing and anti-inflammatory cells (33). Interestingly, while we detected increased frequency of classical (CD14^+^ CD16^-^) and intermediate (CD14^+^ CD16^dim^) monocyte subsets, we observed near complete depletion of non-classical monocytes (CD14^-^ CD16^+^) (**Figure 2E**). However, in keeping with a contrasting effect of cancer on selected myelopoietic alterations induced by SARS-CoV-2 infection, we found that the frequencies of classical and non-classical monocytes in COV/CA were virtually restored to levels similar to those present in HD and CA (**Figure 2E**).

**Figure 3:**
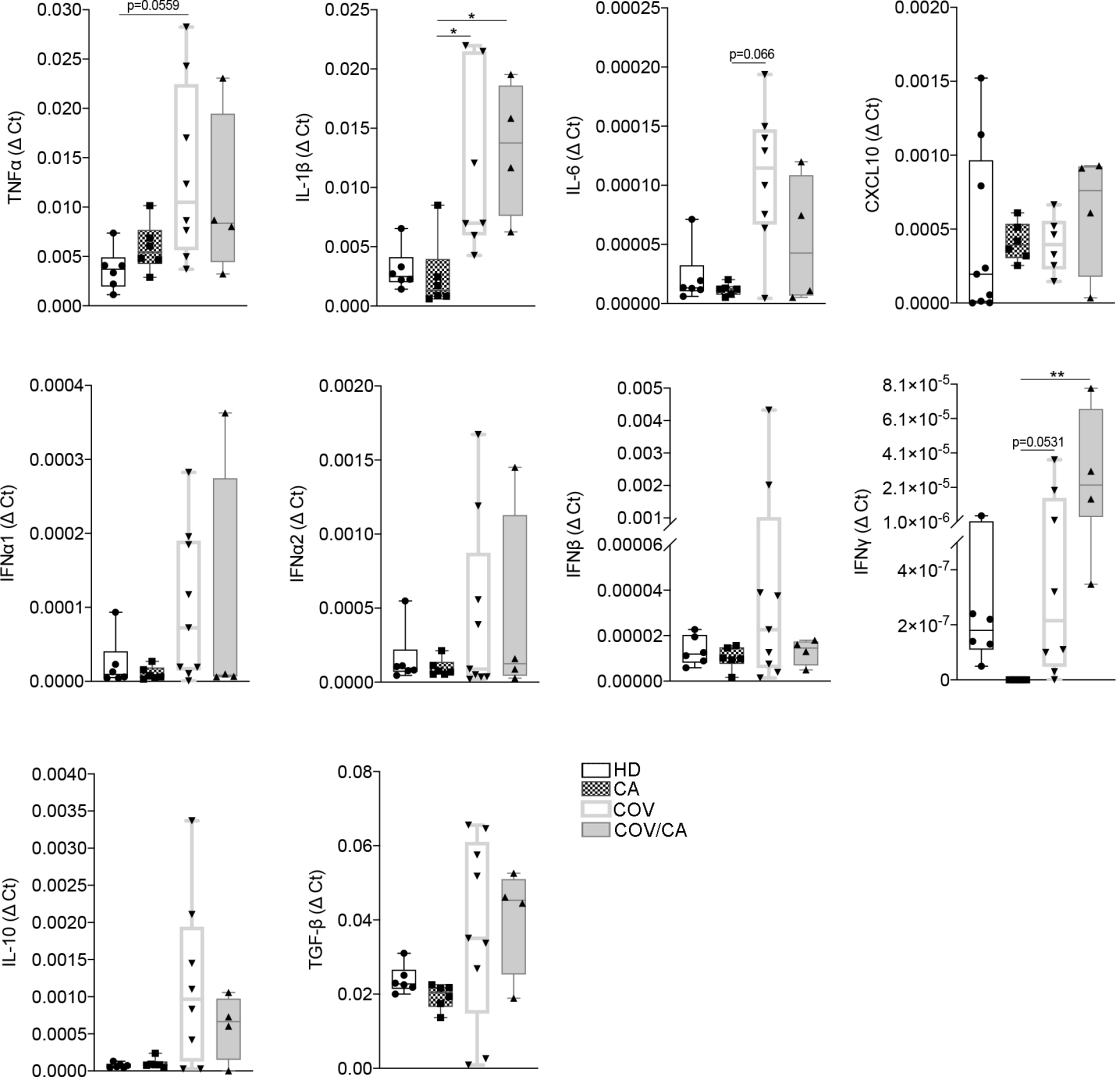
Cytokine gene expression in freshly isolated PBMCs from our study cohorts. Box-and-whisker plots representing mRNA levels of the indicated pro- and anti-inflammatory cytokines in freshly isolated PBMCs from HD (*n* = 6), CA (*n* = 6), COV (*n* = 8) and COV/CA (*n* = 4) patients. Boxplot representation (centre line, mean; box limits, upper and lower quartiles; whiskers, range; points, data points per patient). Statistical significance of differences between patient groups was calculated using one-way ANOVA followed by Tukey’s multiple comparison test or a Kruskal-Wallis test followed by Dunn′s multiple comparisons test. *P < 0.05, **P < 0.01 between selected relevant comparisons.

Next, the mRNA expression levels of key cytokines from freshly isolated PBMCs were determined by RT-PCR (**Figure 3**). Consistent with previous reports (34–37), COVID-19 patients displayed higher levels of *TNFα*, *IL-1β*, *IL-6, IFNα*, *IFNβ*, *TGFβ* and *IL-10* in comparison with HD. Interestingly, in COV/CA vs COV we consistently noticed upregulation of *IFN-γ* and downregulation of IL-6 expression levels (**Figure 3**), further supporting a regulatory effect of cancer on the activation state of circulating leukocyte populations induced by SARS-CoV-2 infection (6).

To confirm this hypothesis, we next undertook a multiomics-based approach and analyzed the immunometabolic state of PBMCs and plasma from our patient cohorts.

**Figure 4:**
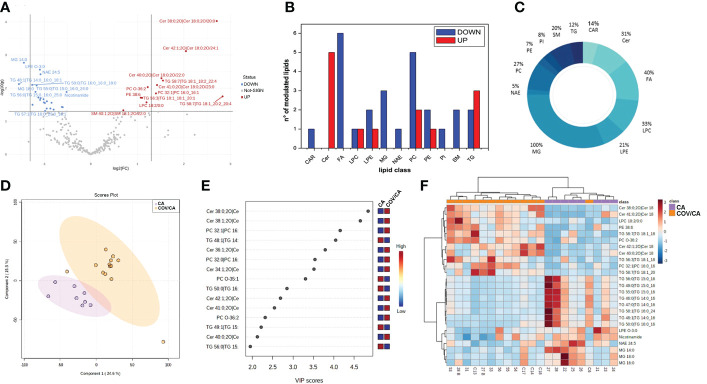
Alteration of lipids in PBMCs from COVID-19 cancer patients vs cancer-only patients. **(A)** Volcano plot depicting the modulation of 35 lipids (FC > 1.3 and *p*-value < 0.05), 23 under-expressed and 12 over-expressed; graphs showing **(B)** the number of upregulated (red) and downregulated (blue) lipid species within each single class of lipids and **(C)** the percentage of modulated lipid species for each lipid class. **(D, E)** Partial least square discriminant analysis (PLS-DA) of PBMC lipidomic profiles, showing **(D)** the presence of two separate groups of patients and **(E)** the most important discriminating variables for each group, colored boxes indicating the most predictive or discriminative features in each group, VIP scores (variable importance in the projection) (red: high; blue: low). **(F)** Hierarchical heat maps of the abundances of quantified lipids highlighting the two clusters of samples, with COV/CA in orange and CA in purple. CA (*n* = 8) and COV/CA (*n* = 13) patients.

*Dysregulation of lipids in PBMCs from COVID-19 cancer patients.* Lipidomic analysis performed on PBMCs from CA, COV and COV/CA patients allowed us to detect and quantify 251 lipid species belonging to the following 17 lipid classes: acylcarnitine (CAR), cholesteryl ester (CE), ceramide (Cer), diacylglycerol (DG), free fatty acid (FA), glycosylceramide (HexCer), lysophophatidylcholine (LPC), lysophosphatidylethanolamine (LPE), lysophosphatidylinositol (LPI), monoacylglycerol (MG), N-acyl ethanolamines (NAE), phosphatidylcholine (PC), phosphatidylethanolamine (PE), phosphatidylinositol (PI), phosphatidylserine (PS), sphingomyelin (SM), and triacylglycerol (TG). Among these, the most abundant classes were PEs, TGs and PCs, each with 43, 41 and 26 identified lipids, respectively. Statistical analysis of lipid abundance showed only six differentially modulated lipids between COV and COV/CA patients, with five being upregulated (DG 32:5, CE 26:6, NAE 22:1, DG 22:1 and NAE 14:1) and only one downregulated (TG 52:1) (**Supplemental Table 1**). By contrast, in COV/CA vs CA we recorded 23 under-expressed and 12 over-expressed lipid species (**Figure 4A**; **Supplemental Table 1**). The global decrease in lipids, including LPCs, LPEs, PCs, PEs and PIs (**Figures 4B–D**), observed in COV/CA vs CA indicates that SARS-CoV-2 infection may have a dominant impact on the lipid composition of PBMC membranes. In this regard, PCs and PEs have been shown to play an important role in the formation of microdomains, which affect the dynamics of cell membrane and transmembrane cell signaling (38, 39). Interestingly, the downregulation of LPC 18:0 and LPE O-18:1 seen in both COV and COV/CA patients (**Supplemental Figure 1A**) correlated with the inflammatory profile of these latter (**Figure 3**). Of note, low levels of LPC 18:0 and LPE O-18:1 have also been reported in the serum from chronic HBV patients (39, 40).

Further analysis revealed a marked decrease in glycerophospholipids in COV/CA patients (**Supplemental Table 1**), characterized by the presence of acyl chains with 18 or 20 carbon atoms and 2 or 4 unsaturation, namely PC 16:0_18:2; PC 18:1_20:4, PC 18:2_20:4 and PE 16:0_20:4. This lipid profile implies a potential release of arachidonic acid (FA 20:4) and linoleic acid (FA 18:2), two well-known precursors of prostaglandins (41). Although these last two fatty acids were not detected in PBMCs, metabolomics analysis showed a higher concentration of arachidonic acid in the plasma of COV/CA vs COV and CA (**Supplemental Figure 3**). Furthermore, the downregulation of glycerophospholipids was probably the result of the lower number of identified N-acylethanolamines (NAEs) (n=20) and their downmodulation in COV/CA (**Supplemental Table 1**). In fact, NAEs are synthesized starting from PEs, PCs and their lysophospholipids, which act as acyl donors, through the action of two enzymes (transacylase and phosphodiesterase) (42).

We also found a substantial downregulation of the following phosphoinositols (PIs): 18:0_20:3 (FC = 0.444; P = 0.014); 36:4 (FC = 0.663; P = 0.0495); 38:4 (FC = 0.624; P = 0.002); and 18:1_20:4 (FC = 0.567; P = 0.0356) (**Supplemental Table 1**). As PIs are lipids able to neutralize the infection with respiratory syncytial virus (RSV) by blocking its attachment to the epithelial cell plasma membrane (43), their downmodulation in COV/CA suggests a potential lack of this prevention mechanism (43).

PBMCs from COV/CA patients were also characterized by higher levels of ceramides (**Figures 4E, F**; **Supplemental Figure 1B**). Ceramides are cone-shaped membrane lipids that, thanks to their hexagonal structure, promote the aggregation of the lipid rafts, leading to the formation of a negative curvature of the cell membrane, which favors viral entry (44, 45). Importantly, recent studies have linked ceramides to SARS-CoV-2 entry into human epithelial cells (46, 47), and increased levels of ceramides have been detected in serum from COVID-19 severe patients (48). Fittingly, we show that ceramide levels correlate with the severity of COVID-19 (**Supplemental Figure 1B**).

The lipidomic analysis also revealed a connection between ceramide levels, NAD metabolism and sirtuins. Indeed, besides the high level of ceramides, we observed decreased concentration of intracellular nicotinamide in PBMCs from COVID-19 patients, which was more accentuated in severe COVID-19 patients (**Figure 4F**; **Supplemental Figure 1C**). In this regard, it is important to consider that nicotinamide is converted to the nicotinamide adenine dinucleotide (NAD) precursor nicotinamide mononucleotide (NMN) by the rate limiting enzyme nicotinamide phosphoribosyltransferase (NAMPT) (49). Furthermore, NAD^+^ plays a crucial role in maintaining cellular energy and tissue homeostasis *via* redox reactions through a range of NAD-dependent enzymes, including sirtuin deacetylase 1 (SIRT1) (6, 50), overseeing both innate immunity and homeostatic robustness of physiological mechanisms (51). Intriguingly, a bidirectional relationship has been suggested between the metabolism of NAD and the protective role that angiotensin-converting enzyme 2 (ACE-2), the SARS-CoV-2 receptor, can play against hyperinflammation (6), while the reduced activity of the NAD-dependent protein deacetylase SIRT1 represents a major driver of frailty in the elderly (52, 53). In agreement, NAD availability also depends on the *de novo* synthesis by the tryptophan/kynurenine pathway, whose reduction during viral infection has been associated with exacerbated inflammation and low CD4^+^ T cell recovery (54).

A link between ceramides and NAD metabolism was first demonstrated by Rahman et al. who found that augmented ceramide levels in a genetically modified Drosophila affected NAD^+^ level and sirtuin activity, leading to NAD reduction (55). As shown in **Supplemental Figure 1C**, the decrease in nicotinamide levels was more accentuated in COV/CA, suggesting that this pathway represents a more vulnerable metabolic node in patients with concomitant viral infection and neoplastic disease. In contrast, increased disease severity correlated with higher ceramide levels (**Supplemental Figure 1B**).

### Proteomic alterations in PBMCs from cancer and COVID-19 patients

**Figure 5:**
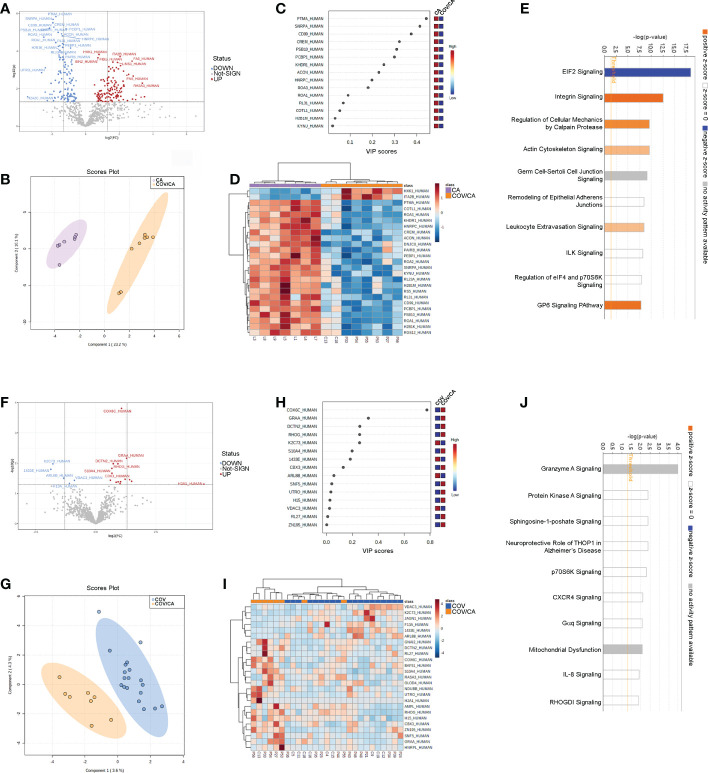
Proteomic characterization of PBMCs in CA vs COV/CA patients. **(A)** volcano plot reporting the modulation of 264 proteins (FC > 1.3 and *p*-value < 0.05), 135 over-expressed and 129 under-expressed proteins; **(B, C)** Sparse partial least square discriminant analysis (sPLS-DA) of PBMC proteomic profiles, showing **(B)** the presence of two separate groups of patients and **(C)** the most important discriminating variables for each group. Colored boxes indicate the most predictive or discriminative features in each group (red, high; blue, low). **(D)** The hierarchical heat maps of the abundances of quantified proteins, highlighting the two clusters of samples, with cancer COVID-19 (COV/CA) patients in orange and cancer (CA) patients in purple. **(E)** Bar-plot presenting the top canonical pathway analysis of significantly altered PBMCs proteins regulated in COV/CA vs CA. **(F–J)** Volcano plot, sparse partial least square analysis, heatmap of protein abundances, and canonical pathway analysis in COV/CA vs COV patients, 15 proteins were over-expressed while 5 were under-expressed. CA (*n* = 7), COV (*n* = 19) and COV/CA (*n* = 8) patients.

To further elucidate the interplay between SARS-CoV-2 infection and cancer, we analyzed the global proteome of PBMCs. Out of 869 proteins quantified, 264 were differentially expressed in COV/CA vs CA (**Figure 5A**; **Supplemental Table 2**), whereas only 20 displayed a different expression pattern in COV/CA vs COV patients (fold change > 1.3, P ≤ 0.05) (**Figure 5F**; **Supplemental Table 2**). Furthermore, sparse partial least squares discriminant analysis (sPLS-DA) of either comparison showed a good separation between the two groups of patients (**Figures 5B, G**). The variable importance values in sPLS-DA are reported in **Figures 5C, H**, highlighting the top 15 important features identified by this analysis. The proteomic clustering results relative to the top 25 regulated proteins (t-test) of the two comparisons are shown in the form of heat maps (**Figures 5D, I**).

Next, we performed canonical pathway analysis to identify the main pathways associated with SARS-CoV-2 infection in cancer patients (**Figures 5E, J**). eIF2 signaling was the most significantly modulated pathway, predicted inactivated, in COV/CA vs CA (**Figure 5E**). This inactivation may constitute the first host defense mechanism against viral infection. In fact, the accumulation of double strand RNAs and viral proteins induces cellular stress and leads to the activation of the two eIF2α kinases PERK and PRK (56, 57). As a result, the phosphorylation of eIF2α induces a global inhibition of the host translational machinery that does not, however, affect virus replication, but rather favors viral protein production. This may in part explain the decreased concentration of non-phosphorylated IF2A (fold-change 0.55) in COV/CA compared to CA (**Supplemental Figure 2D**). Noteworthy, PERK mediates proteasomal degradation of p53 independently of translational control (58), suggesting that SARS-CoV-2 infection may be setting the stage for DNA damage, thus increasing the susceptibility to oncological diseases (59).

The mechanism whereby SARS-CoV-2 avoids the suppression of viral mRNA translation to ensure continuous viral protein production is at present unclear (60). In our study, we found 31 modulated proteins associated with eIF2 signaling. Of these, 24 were downregulated (i.e., IF2A, ACTB, HNRNPA1, RPL15, RPL23, RPL23A, RPL26L1, RPL29, RPL3, RPL30, RPL31, RPL34, RPL4, RPL8, RPS21, RPS4X, RPS5, RPS6, RPS9, RPL19, RPL32, RPL27, RPS17, RPS20), whereas 6 were upregulated (IF2A, EIF3D, HSPA5, RPL24, RPL27A, and RPS3) (**Supplemental Table 2**). Moreover, a significant decrease in ribosomal protein L26 like 1 (RPL26L1) level was associated with disease severity (**Supplemental Figure 2E**). Notably, RPL26L1 was previously identified by affinity proteomics analysis in both SARS-CoV-2 and Zika vRNA-host protein interactomes (61)

Our pathway analysis also revealed a predicted alteration of integrin and leucocyte extravasation signaling in COV/CA vs CA (**Figure 5E**), suggesting a dominant influence of SARS-CoV-2 infection on this inflammatory trait (62–64). Among the proteins involved in integrin signaling (**Supplemental Figures 2A–C**), our data show reduced levels of both beta (β)-actin (ACTB), required for embryonic development and cell recruitment (65), and Ras-related C3 botulinum toxin substrate 2 (RAC2), whose activation has been linked to infantile-onset combined immunodeficiency and susceptibility to viral infections (66). RAC2 is a plasma membrane-associated small GTPase belonging to the Rho family, which also comprises RAC1 and RAC3 (67). While RAC1 and RAC2 share a redundant role at later stages of T-cell development, RAC1 is downregulated in COVID-19 patients with mild symptoms compared to healthy subjects (68). Interestingly, the heterozygous activating mutation of RAC2 leads to infantile-onset combined immunodeficiency as well as viral infection susceptibility (66).

Our analysis also uncovered increased levels of ADP-ribosylation factor 5 (ARF5) in COV/CA vs CA (**Supplemental Figure 2B**). ARF5 is a member of the human ARF gene family, which is involved in cell proliferation, motility and differentiation though regulation of cellular trafficking, cancer cell survival, migration and invasion (12, 69). Noteworthy, NAD-dependent ADP-ribosylation is emerging as an important regulator of innate immunity through modulation of IFN type I and II activity, which is targeted by some viruses to counteract host antiviral mechanisms (70, 71). In COV/CA patients, ARF5 expression was 7.9-fold higher than that observed in CA (**Supplemental Figure 2B**), while beta-actin (ACTB) expression was downregulated by 1.6-fold (**Supplemental Figure 2A**).

**Figure 6:**
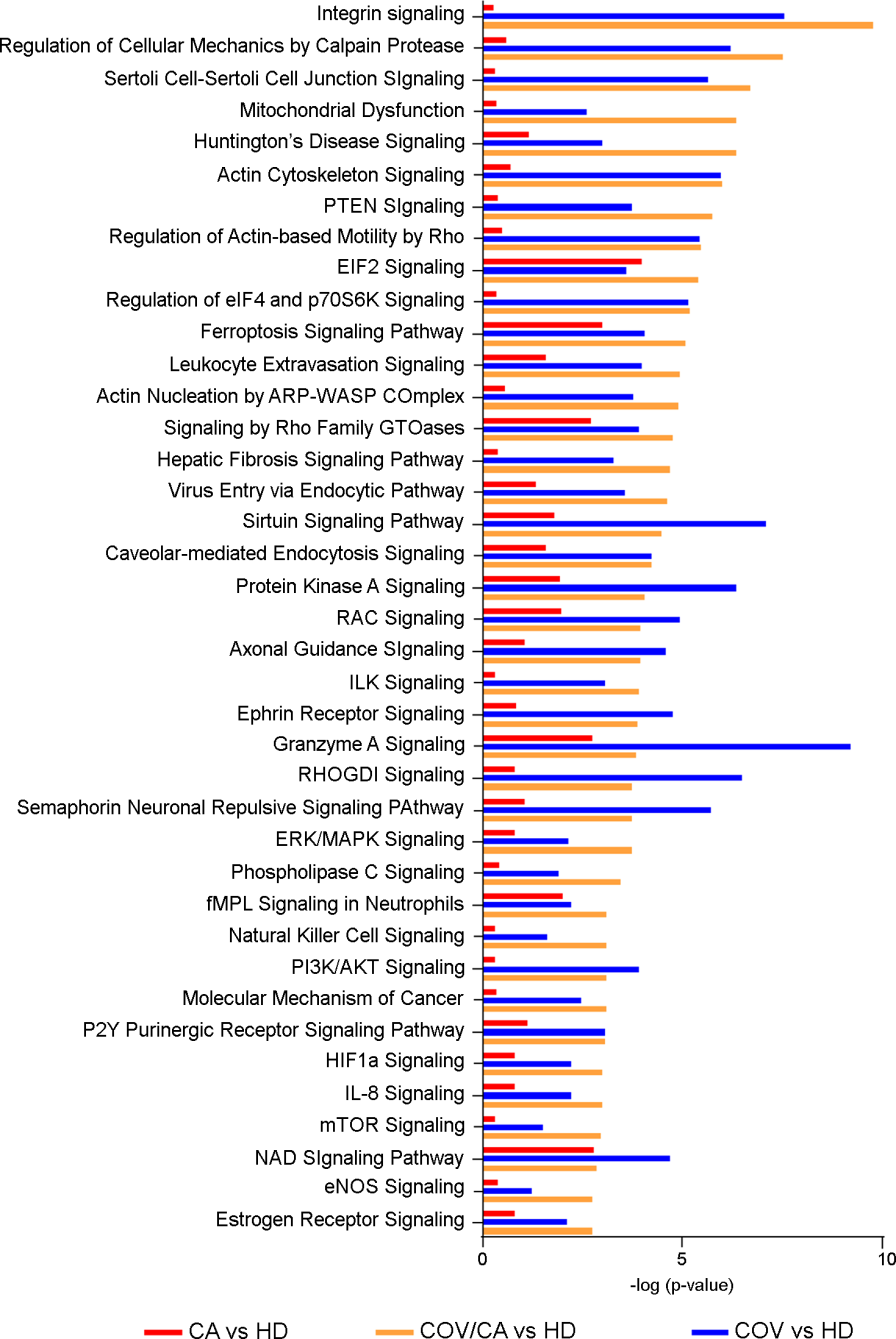
Canonical pathway analysis. Bar-plot presenting the main modulated canonical pathways (-log(p-value)) related to PBMCs proteins in COV/CA (yellow bar), COV (blue bar) and CA (red bar) compared to healthy subjects.

We next compared the significance (-log[p-value]) (**Figure 6**) and prediction (z-score) (**Supplemental Table 5**) of all the canonical pathways evidenced by the proteomic-bioinformatics analysis in order to identify the main pathways involved in the immunometabolic changes observed in our study population. Intriguingly, we found that the marked inhibition of both NAD and sirtuin signaling in COV patients (z-score = -1.9 for NAD and -2.1 for sirtuin) was alleviated in COV/CA patients (z-score = -1.34 for NAD and -0.38 for sirtuin), supporting the hypothesis that cancer can counteract SARS-CoV-2-induced inflammation by enhancing the intracellular nicotinamide phosphoribosyltransferase (NAMPT)-NAD^+^-sirtuin-1 (SIRT1) cascade (6). SIRT1, a NAD-dependent protein deacetylase, plays regulatory roles in different cellular processes such as chromatin structure, gene transcription, metabolism, circadian rhythm, and inflammation (72). The upregulation of NAMPT expression in response to microbial moieties or inflammatory cytokines (i.e. IFNγ) leads to NAD-dependent activation of SIRT1 deacetylase, which limits inflammation and restores tissue homeostasis (73). Interestingly, our data suggest that the predicted inactivation of SIRT1 signaling may also be linked to eIF2a signaling inactivation, whose alteration may result in slower post-stress translation recovery. In this regard, Ghosh et al. (74) demonstrated that SIRT1 regulates eIF2a phosphorylation by forming a complex with two mediators of its dephosphorylation, GADD34 and CReP, suggesting a role of SIRT1 in maintaining the steady-state level of phospho-eIF2a.

Another interesting result of our analysis is the predicted activation of estrogen receptor (ER) signaling in both COV/CA (z-score=2.23) and COV patients (z-score=1.34) compared to HD. This observation is in line with the emerging notion that ER signaling, involved in innate and adaptive immunity, plays a role in SARS-CoV-2 infection (75). Furthermore, in good agreement with the observation that both estrogen and ERs contribute to the activation and proliferation of T-lymphocytes and lead to high expression of IFNγ (76), we found increased expression of IFNγ in PBMCs from COV/CA vs COV patients (**Figure 3**). Interestingly, experimental studies in SARS-CoV-2-infected mice have shown how ovariectomy or treatment with an estrogen receptor antagonist can increase the death rate of this animals, suggesting that ER signaling may play a protective role against severe cases of SARS-CoV-induced inflammation (77).

With regard to cell survival pathways, we detected a marked activation of the phosphatidyl-inositol-3-kinase (PI3K)/AKT cascade in COV vs HD. This signaling pathway, which regulates different aspects of cell survival, such as protein synthesis, apoptosis inhibition and cell proliferation, has been recently linked to blood clot generation. In particular, Pelzl et al. [26] have shown that PI3K/AKT phosphorylation is significantly associated with platelet activation in severe COVID-19 patients, suggesting that the inhibition of PI3K/AKT phosphorylation may be a promising strategy to prevent the onset of thrombosis in COVID-19 patients.

COVID-19 can induce hypoxemia and overexpression of hypoxia-inducible factor-1α (HIF-1α) (78), which is involved in the genesis, angiogenesis, invasion and metastasis of lung cancer (79). However, the modulation of HIF1α signaling was more evident in COV/CA vs HD than that seen in COV vs HD (**Figure 6**).

### Cancer strongly affects the plasma metabolic profile of COVID-19 patients

In order to identify plasma alterations associated with the observed changes in circulating immune cells (**Figures 1**, **2**), we performed untargeted metabolomics analysis on plasma samples from the different patient cohorts. Metabolomic analysis was carried out including all the samples previously investigated by cytofluorimetry, proteomic and lipidomic analysis, obtaining a complete multilevel characterization of each single patient. Twenty-one additional subjects (10 HD and 11 CA), all with a negative RT-PCR test for SARS-CoV-2 infection, were included in this analysis. More than 230 plasma molecules with significant modulations were identified and quantified across the different patient cohorts (**Supplementary Table 3**).

**Figure 7:**
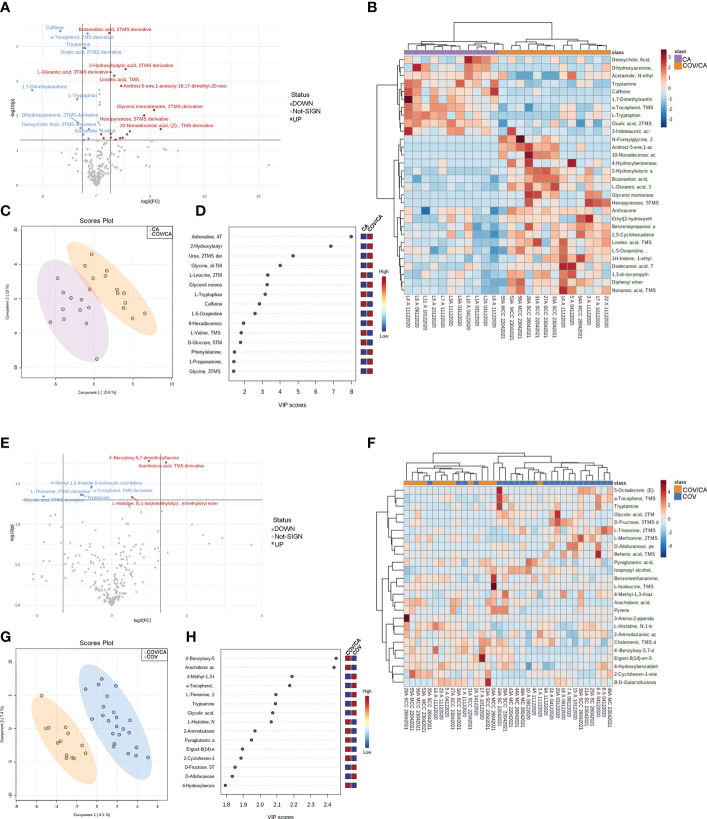
Plasma metabolome in cancer patients with or without COVID-19. **(A)** volcano plot depicting the modulation of 230 small molecules quantified (FC > 1.3, *p* ≤ 0.05), 25 small molecules were modulated, of which 16 up-regulated and 10 down-regulated. **(B)** Hierarchical heat maps of levels of quantified molecules, highlighting the two clusters of samples, with COV/CA in orange and CA in purple. **(C, D)** Partial least square discriminant analysis (PLS-DA) of metabolome profiles, showing **(C)** the presence of two separate groups of patients and **(D)** the most important discriminating variables for each group. **(E–H)** Volcano plot, heatmap of molecule levels and PLS-DA of COV/CA vs COV, 5 small molecules were down regulated while 3 increased their levels. CA (*n* = 11), COV (*n* = 23) and COV/CA (*n* = 13) patients.

We first focused on the differences between COV/CA and CA (**Figure 7**). PLS-DA revealed the presence of several metabolomic signatures discriminating COV/CA from CA (**Figure 7C**), while volcano plotting (**Figure 7A**) identified the molecules with the most significant changes between the two groups (fold change > 1.3 and p-value < 0.05). To obtain an overview of the main changes, we performed heatmap and hierarchical clustering analysis using abundances of the 40 metabolites with the lowest p-values (p < 0.05) in COV/CA vs CA (**Figure 7B**) and the top 25 metabolites in COV/CA vs COV (**Figure 7F**). **Figures 7E, H** show the volcano plot and the most discriminating variables in COV/CA vs COV patients, while **Figure 7G** highlights the existence of distinct metabolomic signatures between these two cohorts.

**Figure 8:**
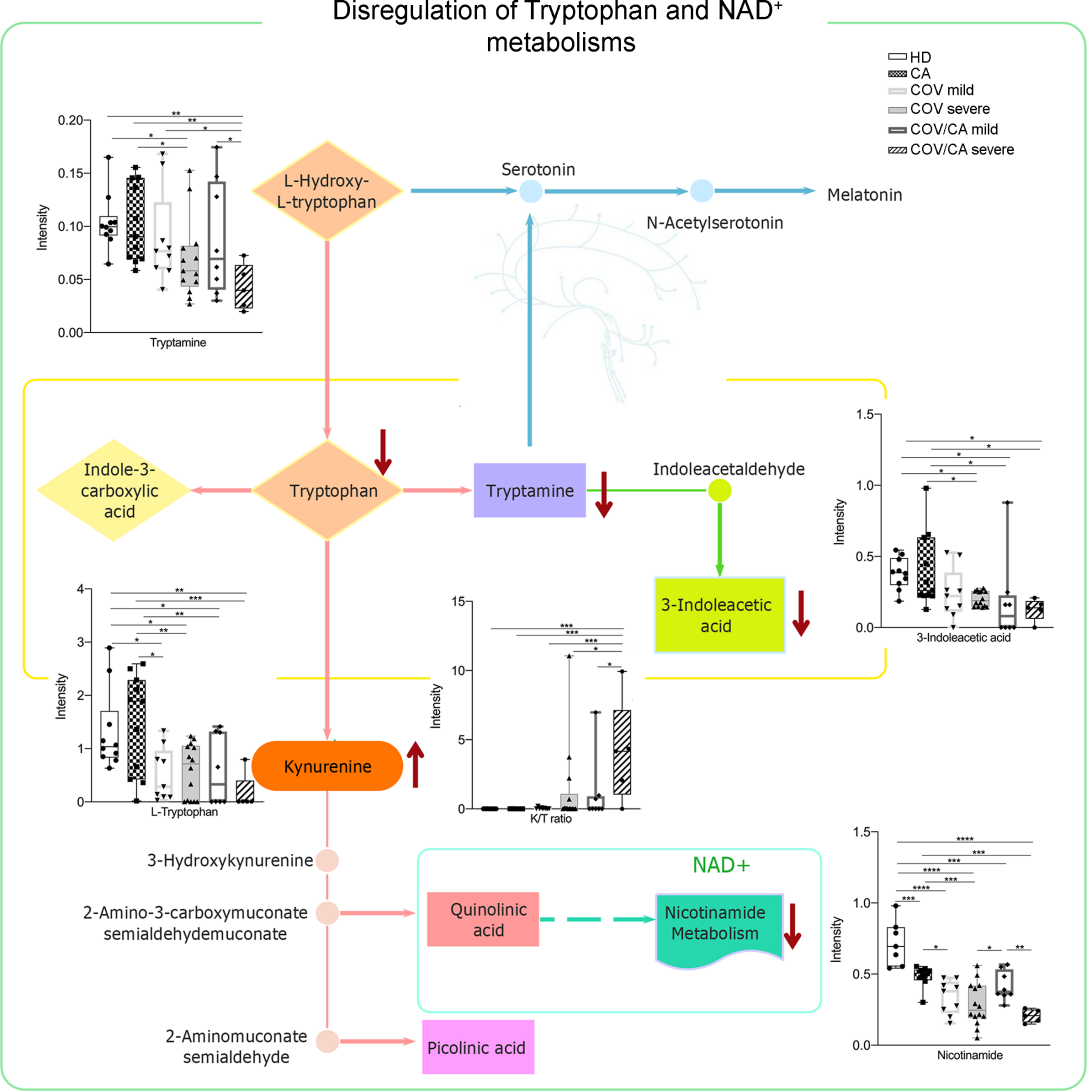
Biochemical map of plasma dysregulations of Tryptophan and NAD^+^ metabolism. Boxplots of the main altered molecules: tryptamine, L-tryptophan, 3-indoleacetic acid, nicotinamide and K/T (kynurenine/L-tryptophan) ratio. Healthy subjects (green), cancer patients (purple), cancer patients with (orange) or without (blue) COVID-19; *P < 0.05, **P < 0.01, ***P < 0.001, ****P < 0.0001.

As shown in **Figure 8**, COV/CA patients were characterized by lower levels of tryptophan, tryptamine and 3-indoleacetic acid, as compared to both CA and HD groups. This downmodulation correlated with a marked reduction in nicotinamide levels in COV patients, regardless of the presence of cancer (**Figure 8**), suggesting that a strong perturbation of both tryptophan and NAD metabolism takes place during the evolution of COVID-19. Nevertheless, the downregulation of plasma tryptamine (**Figure 8**) and α-tocopherol (**Supplemental Figure 3**) was less pronounced in COV than COV/CA patients, indicating a cancer-dependent metabolic specificity. This inference was further supported by the strong increase in the tryptophan/kynurenine ratio observed in COV/CA patients (**Figure 8**). Conversely, while glycine, linoleic acid and phenylalanine were increased in COV patients either with or without cancer, L-glutamic acid, succinic acid and the intermediate of amino acid catabolism α-hydroxybutyrate seemed to be more pronounced in oncological patients with severe COVID-19 (**Supplemental Figures 3G, H, J**, respectively) compared to COV patients. Combined ROC curves of most relevant small molecule biomarkers were also reported in **Supplemental Figure 4**. The combined ROC curve (Oxalic acid and alpha-Tocopherol) for the comparison between CA and CA/COV group was characterized by an AUC of 0.971, the combined ROC curve (Arachidonic acid and alpha-Tocopherol) that better discriminate Covid-19 patients from Covid-19 patients with cancer reported an AUC of 0.78, while the Combined ROC (c) of the five molecules (alpha-Tocopherol, 3-Indoleacetic acid, Arachidonic acid, Tryptamine and L-Tryptophan) of the comparison between healthy subject and Covid-19 patients showed an AUC of 0.977 (**Supplemental Figures 4A–C**, respectively).

**Figure 9:**
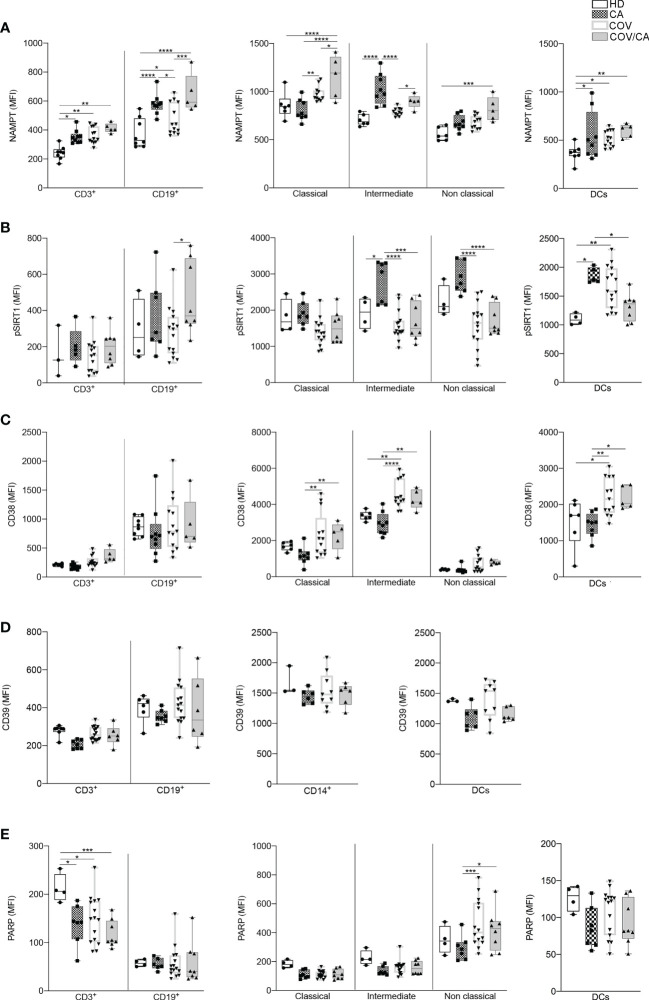
Flow cytometry analysis of NAMPT and NAD-dependent enzymes in the different patient cohorts. Box-and-whisker plots representing FACS quantification of **(A)** NAMPT, **(B)** pSIRT1, **(C)** CD38, **(D)** CD39 and **(E)** PARP expression in the indicated immune cell populations (CD3^+^ T cells, CD19^+^ B cells, DCs and monocytes subsets). Samples were freshly isolated PBMCs from HD (*n*=8), CA (*n*=9), COV (*n*=13) and COV/CA (n=5) patients. Boxplot representation (centre line, mean; box limits, upper and lower quartiles; whiskers, range; points, data points per patient). **(A)** HD (*n* = 6 or 8), CA (*n* = 8), COV (*n* = 12 or 13) and COV/CA (*n* = 5) patients. **(B)** HD (*n* = 3 or 4), CA (*n* = 5 or 7), COV (*n* = 15) and COV/CA (*n* = 8) patients. **(C)** HD (*n* = 6), CA (*n* = 8), COV (*n* = 13) and COV/CA (*n* = 5) patients. **(D)** HD (*n* = 3 or 4), CA (*n* = 6 or 7), COV (*n* = 9 or 15) and COV/CA (*n* = 6) patients. **(E)** HD (*n* = 4), CA (*n* = 6 or 7), COV (*n* = 15) and COV/CA (*n* = 8) patients. Statistical significance of differences between patient groups was calculated using two-way ANOVA with Sidak’s multiple comparisons test. *P < 0.05, **P < 0.01, ***P < 0.001, ****P < 0.0001 between selected relevant comparisons.

Based on the well-established immunosuppressive activity of tumors (80) and their ability to induce alternative/M2 inflammation programs (7), the results from our metabolomic analysis indicate that tumors can interfere with selected metabolic alterations induced by SARS-CoV-2 infection. In support to this hypothesis, we found higher levels of phospho-SIRT1 (pSIRT1) in both intermediate and non-classical monocytes from CA vs COV patients (**Figure 9B**, center). Of note, as compared to COV, COV/CA patients also displayed enhanced NAMPT levels in all monocyte subsets (**Figure 9A**, center) as well as in CD3^+^ T cells and CD19^+^ B lymphocytes (**Figure 9A**, left). Additionally, restoration of pSIRT1 was observed in CD19^+^ B lymphocytes from COV/CA patients (**Figure 9B**, left). Finally, COV/CA DCs displayed sustained levels of the NAD^+^-consuming CD38 enzyme, a marker of activation and maturation (81) (**Figure 9C**, right). In contrast, no significant changes were observed in the expression of the NAD^+^-consuming enzymes CD39 (**Figure 9D**) and PARP (**Figure 9E**) in COV vs COV/CA. Noteworthy, the nicotinamide deficiency observed in COV/CA patients (**Supplemental Figure 1C**), potentially exacerbated by CD38 overexpression (**Figure 9C**), may affect NAD^+^ availability and consequently key physiological processes (51).

In order to rescue NAD^+^ levels, we treated PBMCs purified from HD, CA, COV and COV/CA patients with a CD38 inhibitor (apigenin) and NAD^+^ precursors (leucin and nicotinamide). As shown in **Supplemental Figure 5**, despite with different amplitude, 1h treatment with nicotinamide successfully increased NAD^+^ levels respect to the relative untreated sample, whereas stimulation with apigenin and leucine had no effect. Nevertheless, nicotinamide-induced increment of intracellular NAD^+^ level in COV and COV/CA- patient did not bring them back to physiological healthy levels, which was about 4 folds higher (not shown). These results indicate that rescuing NAD+ pathway, might be beneficial to COV patients and particularly for COV/CA patients.

## Discussion

The molecular basis underlying the severity of COVID-19, in either its acute or long-term phase, in cancer patients is poorly known. The need to gain insights into the interplay between cancer and COVD-19 becomes even more compelling when we consider the frequency of long COVID-19 symptoms detected in the infected population and their possible involvement in chronic damages.

To fill this gap, in this study we have performed flow cytometric and multiomics analyses of blood samples obtained from COVID-19 patients, with or without neoplastic disease. While cancer and COVID-19 share an elevated risk of thrombosis, with increased D-dimer levels (82), we show that the co-existence of neoplasia and COVID-19 inhibits the upregulation of the inflammatory biomarkers CRP and D-dimer, notoriously associated with COVID-19 severity (1, 19, 20), suggesting that the reduced expression of IL-6 observed in COV/CA PBMCs may result in reduced tissue factor levels (83) and thus in the concomitant reduction of D-dimer levels.

This mitigating action performed by cancer appears to some extent consistent with the ability of tumors to promote alternative (M2) macrophage activation and, therefore, to antagonize M1 polarized inflammation, typically mounting during the cytokine storm in severe COVID-19 cases (84). This corrective action of cancer on SARS-CoV-2-related inflammation does not seem to be unidirectional as COV/CA patients display enhanced activation of HLA-DR^+^CD38^+^CD4^+^ T cells, and increased IFNγ expression in PBMCs. This apparent paradox implies that the overwhelming and persistent inflammation caused by SARS-CoV-2 infection may lead to T-cell exhaustion, as observed in severe sepsis (85) and chronic viral infection (86). In support of this scenario, there is a common consensus in the literature on the increased vulnerability and susceptibility of cancer patients, exhibiting a strong immunosuppressive state, to contracting the infection (87, 88), while an interaction between commensals and pathogenic microbes and host immunity is thought to impact cancer-related inflammation and immunotherapy (89, 90).

Omics analysis confirmed the influence of cancer on the immunometabolic profile induced by SARS-CoV-2. In particular, while viral infection appears to have a dominant impact on the lipid composition of PBMC membranes, the presence of cancer induces a peculiar lipid profile, with a higher content of arachidonic acid in the plasma of COV/CA patients, as well as of ceramide known to be involved in SARS-CoV-2 entry into human epithelial cells (46, 47, 91). In line with a previous report (55), the increase in ceramide was paralleled by decreased concentration of intracellular nicotinamide, which was even more pronounced in PBMCs from severe COV/CA cases.

The proteomic analysis confirms the predominant influence of SARS-CoV-2 infection over cancer, with 264 differentially expressed proteins in COV/CA vs CA. Among the various signaling cascades analyzed, the phospho-eIF2-dependent pathway was the most heavily downregulated one, suggesting its potential role in favoring viral growth through the inhibition of the host translational machinery (56, 57) as well as in promoting genomic instability through proteasome-dependent degradation of p53 (58). If this is hypothesis is confirmed by functional studies *in vivo*, it would probably lead to a new understanding of the multifaceted interplay between COVID-19 and cancer patients.

Another important observation of our proteomic analysis is the downregulation of the NAD^+^-SIRT1 signaling pathways in COV/CA vs COV, which was evidenced by comparing the significance of the canonical pathway to the prediction obtained from all our proteomic and bioinformatics analyses. In this regard, the overall nicotinamide deficiency observed in COV/CA could be further exacerbated by the overexpression of CD38 observed in myeloid and lymphoid cells, thereby significantly affecting NAD+ availability and related physiological processes (6).

In summary, the metabolic scenario that we observed in COV/CA and COV patients finds a correlation in the analysis of the PBMC phenotype. Indeed, we confirmed that COVID-19 patients display a drop in the percentage of CD3+, CD4+ and CD8+ T cells and monocytes, while indicating that the decrease frequency of cDCs and pDCs may suggest a limited activation of antiviral T and NK cell functions (24). Concomitantly, as compared to COV patients, we report that COV/CA patients have higher frequency of activated HLA-DR+ and CD38+/HLA-DR+ T cells, which is in line with enhanced COVID-19 severity (29). The contrasting effect of cancer on selected myelopoietic alterations induced by SARS-CoV-2, was further supported by the restored frequency of classical and non-classical monocytes in COV/CA, as well as neutrophils, all reverting to levels similar to those present in HD and CA. The role of the low availability of nicotinamide adenine dinucleotide (NAD+), as observed in COV/CA patients, on the PBMC phenotype, is also suggested by the increased expression of NAMPT in their monocytes, CD3+ T lymphocytes and CD19+ B lymphocytes, the latter also characterized by the increase of pSIRT1. Accordingly, the sustained levels of the NAD+-consuming CD38 enzyme that we observed in DCs and lymphocytes may likely affect the NAD+ availability and consequently key physiological processes, resulting in host immune alterations. Indeed, as nicotinamide mononucleotide is a key intermediate of NAD, which plays a fundamental role in the NAMPT/NAD^+^/SIRT1 homeostatic pathway (55), it is reasonable to assume that a balanced nutritional supply of tryptophan, which together with nicotinamide (vitamin B3) provides the primary and rescue pathways for the synthesis of NAD (6), would be beneficial to COV/CA patients. The lower levels of tryptophan, tryptamine and nicotinamide found in the plasma of COV/CA patients, as well as the parallel increase in tryptophan/kynurenine ratio, further point to the NAD pathway as a highly vulnerable metabolic node in these patients.

Although our work is not fully supported by functional evidence and is based on a relatively small number of patients, the data produced indicate that the emerging immunometabolic traits may have potential relevance for the stratification and treatment of patients with severe disease, and indicates the possibility of optimizing patients’ nutritional supplementation (i.e. NAD precursors) in order to strengthen their immunometabolic homeostasis.

Although our study suffers from some limitations, mainly the mixed phenotypes of the tumors and the small size of the analyzed sample, it highlights for the first time with an integrated analysis the immunometabolic interferences between cancer and COVID-19. The data obtained with this approach however highlight how tumors and viruses elicit partially contrasting effects on immune responses, laying the foundations for further investigations aimed at characterizing the mechanisms underlying this interference and its clinical significance

## Data availability statement

The datasets presented in this study can be found in online repositories. The names of the repository/repositories and accession number(s) can be found below: PXD040683 (ProteomeXchange).

## Ethics statement

The studies involving human participants were reviewed and approved by Humanitas Clinical and Research Center Ethics Committee (study number 2490; identification 1366); Italian Ministry of Health (Nos. 97/2014-PR and 25/2018-PR. The patients/participants provided their written informed consent to participate in this study.

## Author contributions

FC, BD, MM, AB and CP are co-first authors and contributed equally to this work. The order of these authors has been established according to seniority. FC performed the cytofluorimetry study and related analyses. BD organized and collected patient biological samples and integrated flow cytometric analyses. MM performed the omics studies. AB performed gene expression analyses. CP coordinated the collection of biological samples for omics analyzes. VG contributed to experiments. VV performed the bioinformatics analyses of omics studies. EM provided critical discussion of the experimental data obtained through the omics studies. EB performed the omics analyses. BB contributed by critical discussions on the experimental results. SB, IM, GC evaluated the clinical profile of the patients, stratifying them in the cohorts used in the study. VT performed the statistical analyses. AS provided the guiding hypothesis of the study and contributed to the experimental design and supervision of the study. All authors approved the submitted version.

## Supplementary material

Supplementary Table 1 List of modulated lipids in PBMCs with fold changes (FC) and *p*-values.

Supplementary Table 2 List of modulated proteins in PBMCs with fold changes (PC) and *p*-values.

Supplementary Table 3 List of identified and modulated small molecules in plasma with fold changes (FC) and *p*-values.

Supplementary Table 4 Tumor types in COV/CA and CA patients.

Supplementary Table 5 Prediction score of the canonical pathway analysis performed on proteomic data.

Supplementary Figure 1 Modulation of lipids and NAD in COVID-19 patients. **(A)** Box-plot plot of LPC 18:0 (left) and LPE O-18:1 (right) concentration in healthy subjects HD), cancer patients (CA), mild and severe COVID-19 patients (COV), and mild and severe patients with COVID-19 and cancer (COV/CA). **(B)** Box-plot plot of total ceramide concentration). **(C)** Box-plot plot of the concentration of the nicotinamide adenine dinucleotide (NAD) precursor nicotinamide. **P* < 0.05, ***P* < 0.01, ****P* < 0.001, *****P* < 0.0001.

Supplementary Figure 2 Box-plot plot of proteins involved in the main pathways modulated in cancer patients with COVID-19. Integrin signaling: actin **(A)**, ADP-ribosylation factor 4 **(B)**, Ras-related C3 botulinum toxin substrate 2 **(C)**. EIF2 signaling: growth factor receptor-bound protein 2 **(D)**, 60S ribosomal protein L26-like 1 **(E)**. NAD signaling: histone H2B type 1-K, H2B K **(F)**, histone H2B type 1-D **(G)**. Sirtuin signaling: isocitrate dehydrogenase [NADP], mitochondrial **(H)**, succinate dehydrogenase [ubiquinone] iron-sulfur subunit, mitochondrial **(I)**. Leucocyte extravagation signaling: CD99 antigen **(J)**. **P* < 0.05, ***P* < 0.01, ****P* < 0.001, *****P* < 0.0001

Supplementary Figure 3 Box-plot of DL-phenylalanine **(A)**, linoleic acid **(B)**, glycerol monostearate **(C)**, pipecolic acid **(D)**, L-threonine **(E)**, oxalic acid **(F)**, 2-hydroxybutyric acid **(G)**, succinic acid **(H)**, alpha-tocopherol **(I)**, L-glutamic acid **(J)**, L-glutamine **(K)**, glycine **(L)**, arachidonic acid **(M)** in HD, CA, mild and severe COV and mild and severe COV/CA patients.; **P* < 0.05, ***P* < 0.01, ****P* < 0.001, *****P* < 0.0001.

Supplementary Figure 4 Combined ROC of most relevant small molecule biomarkers. **(A)** Combined ROC of Oxalic acid and alpha-Tocopherol for the comparison between CA and COV/CA group; **(B)** Combined ROC of Arachidonic acid and alpha-Tocopherol for the comparison between COV and COV/CA patients; **(C)** Combined ROC of alpha-Tocopherol, 3-Indoleacetic acid, Arachidonic acid, Tryptamine and L-Tryptophan for the comparison between HD and COV patients.

Supplementary Figure 5 Intracellular NAD^+^ level variations express as fold change over the relative control in HD, CA, COV and COV/CA patients. Isolated PBMCs were cultured in RPMI media or stimulated with 100 µM Apigenin or 500 µM Leucine or 800 µM Nicotinamide for 1 h before intracellular NAD^+^ quantification. HD (*n* = 11), CA (*n* = 6), COV (*n* = 16) and COV/CA (*n* = 5) patients. Statistical significance of differences between patient groups was calculated using Two-way ANOVA with Sidak’s multiple comparisons test or one-way ANOVA followed by Tukey’s multiple comparison test. **P* < 0.05, ***P* < 0.01, ****P* < 0.001, *****P* < 0.0001 between selected relevant comparisons.

Supplementary Figure 6
**(A)** Representative flow cytometry gating strategy for the identification of major immune cell populations in blood. **(B, C)** Leucocyte gating strategy for phenotypic characterization of lymphocytes **(B)** and monocytes in PBMCs **(C)**.
