# Supplementary material for: Immunometabolic interference between cancer and COVID-19
Source: Front Immunol. 2023 Mar 29;14:1168455. doi: 10.3389/fimmu.2023.1168455 (PMC10090695; doi:10.3389/fimmu.2023.1168455)
Supplement: Supplementary Table 4 — Tumor types in COV/CA and CA patients. [file Table_4.pdf]

COV/CA	CA
Esophageal adenocarcinoma	Lung cancer stage IA
Lung cancer	Lung cancer stage IIA-B
Colorectal cancer	Lung cancer stage IIIA
Renal cell carcinoma	Lung cancer stage IV
Urothelial carcinoma	
Meningioma (prior non-Hodgkin's)	
Colon cancer (prior prostate)	
Pulmonary microcytoma, prostate adenocarcinoma	
Squamous-cell lung carcinoma	
Prostate adenocarcinoma	
Gastric cancer	
Esophageal cancer	
Prostate cancer	
Non-Hodgkin's lymphoma	
Follicular lymphoma	
Gastric adenocarcinoma with peritoneal localization	
Lung adenocarcinoma	
Prostate metastatic cancer	
Rectal adenocarcinoma	
Pancreatic neuroendocrine tumor	
Ovarian neoplasm relapse	
Duodenum neuroendocrine metastatic tumor	
Diffuse large B cell lymphoma	
Acute leukemia ambiguous lineage	
