# Supplementary figures and images for: Immunometabolic interference between cancer and COVID-19
Source: Front Immunol. 2023 Mar 29;14:1168455. doi: 10.3389/fimmu.2023.1168455 (PMC10090695; doi:10.3389/fimmu.2023.1168455)

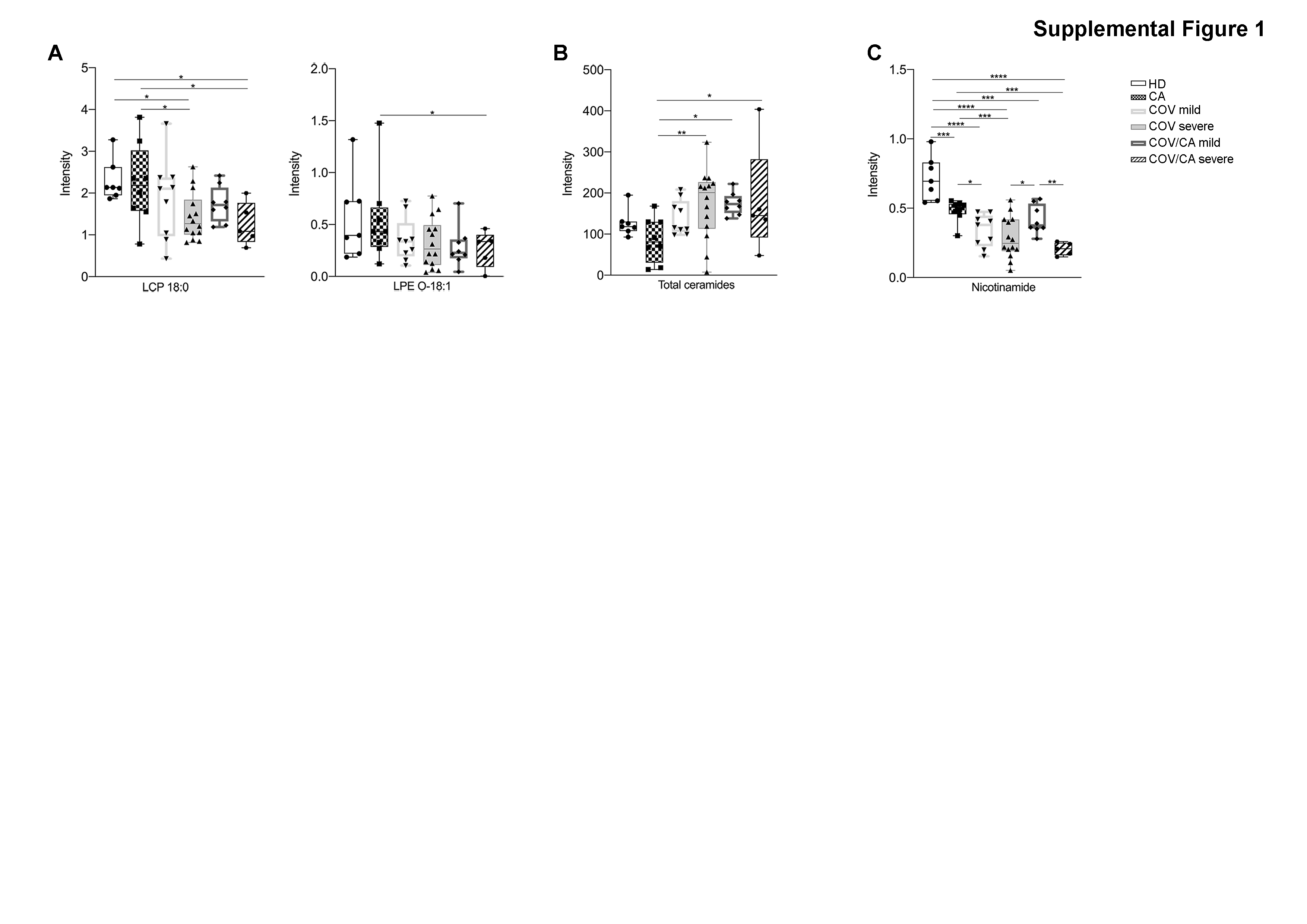

Supplement: Supplementary Figure 1 — Modulation of lipids and NAD in COVID-19 patients. (A) Box-plot plot of LPC 18:0 (left) and LPE O-18:1 (right) concentration in healthy subjects HD), cancer patients (CA), mild and severe COVID-19 patients (COV), and mild and severe patients with COVID-19 and cancer (COV/CA). (B) Box-plot plot of total ceramide concentration). (C) Box-plot plot of the concentration of the nicotinamide adenine dinucleotide (NAD) precursor nicotinamide. *P < 0.05, **P < 0.01, ***P < 0.001, ****P < 0.0001. [file Image_1.tif]

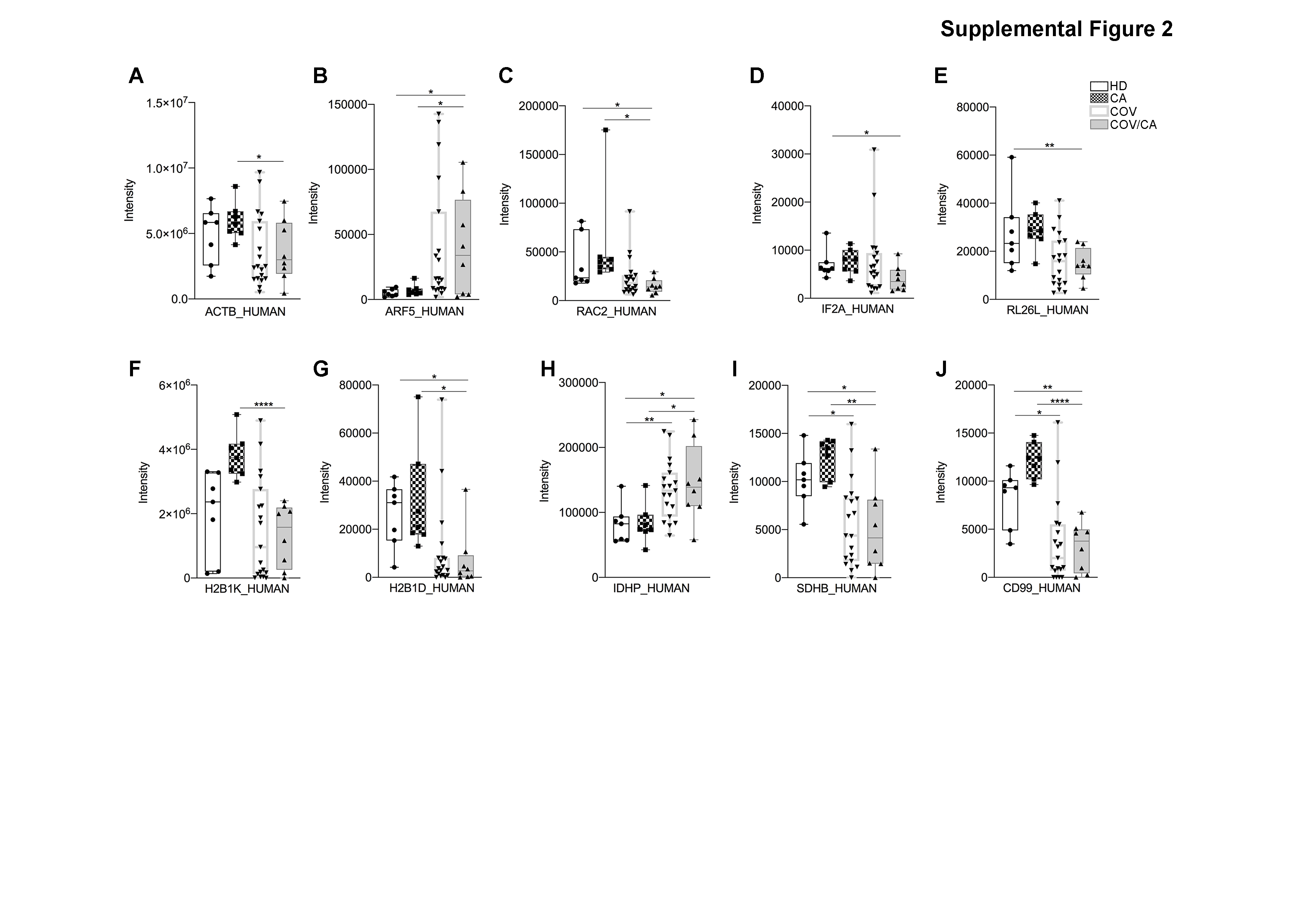

Supplement: Supplementary Figure 2 — Box-plot plot of proteins involved in the main pathways modulated in cancer patients with COVID-19. Integrin signaling: actin (A), ADP-ribosylation factor 4 (B), Ras-related C3 botulinum toxin substrate 2 (C). EIF2 signaling: growth factor receptor-bound protein 2 (D), 60S ribosomal protein L26-like 1 (E). NAD signaling: histone H2B type 1-K, H2B K (F), histone H2B type 1-D (G). Sirtuin signaling: isocitrate dehydrogenase [NADP], mitochondrial (H), succinate dehydrogenase [ubiquinone] iron-sulfur subunit, mitochondrial (I). Leucocyte extravagation signaling: CD99 antigen (J). *P < 0.05, **P < 0.01, ***P < 0.001, ****P < 0.0001 [file Image_2.tif]

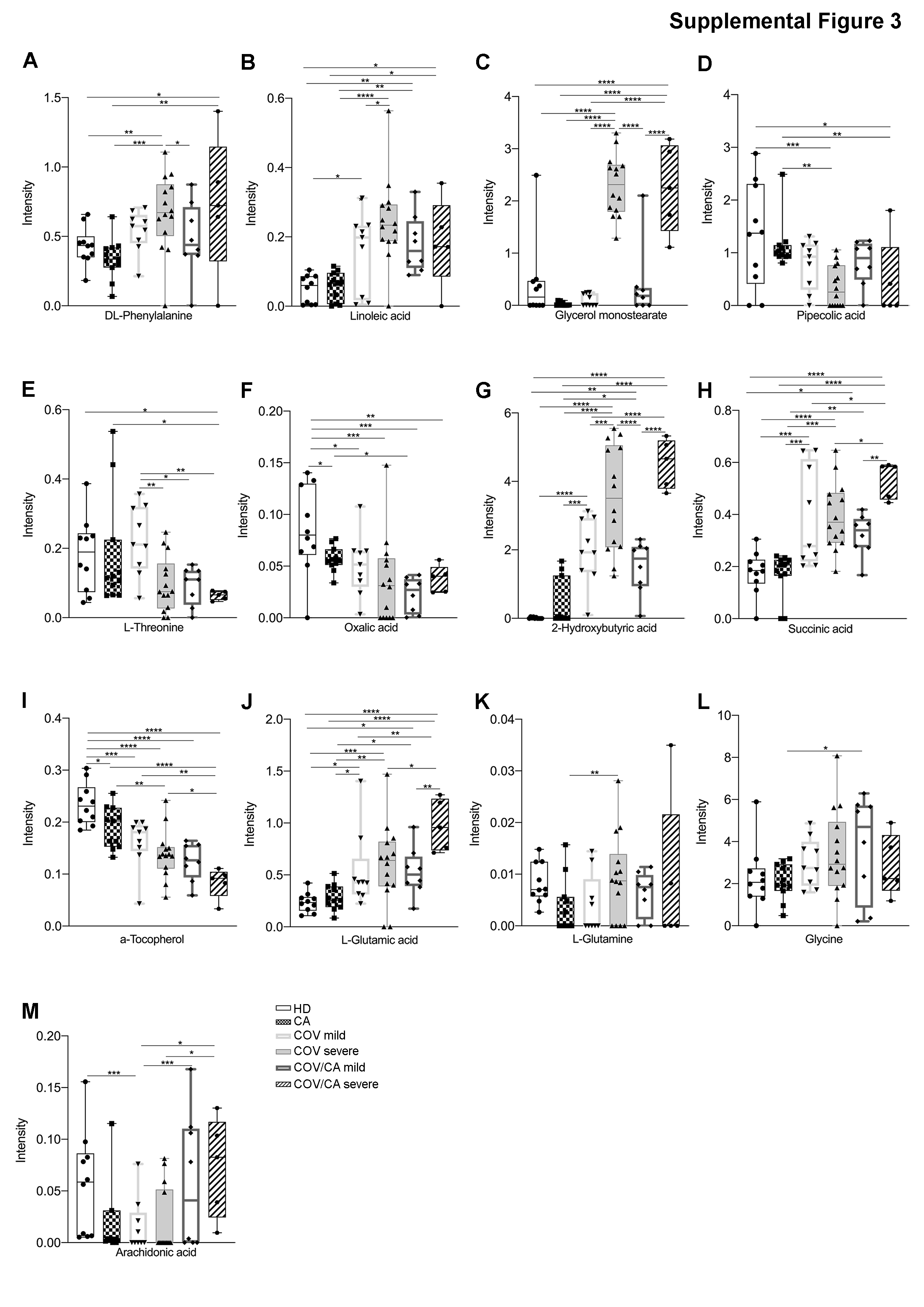

Supplement: Supplementary Figure 3 — Box-plot of DL-phenylalanine (A), linoleic acid (B), glycerol monostearate (C), pipecolic acid (D), L-threonine (E), oxalic acid (F), 2-hydroxybutyric acid (G), succinic acid (H), alpha-tocopherol (I), L-glutamic acid (J), L-glutamine (K), glycine (L), arachidonic acid (M) in HD, CA, mild and severe COV and mild and severe COV/CA patients.; *P < 0.05, **P < 0.01, ***P < 0.001, ****P < 0.0001. [file Image_3.tif]

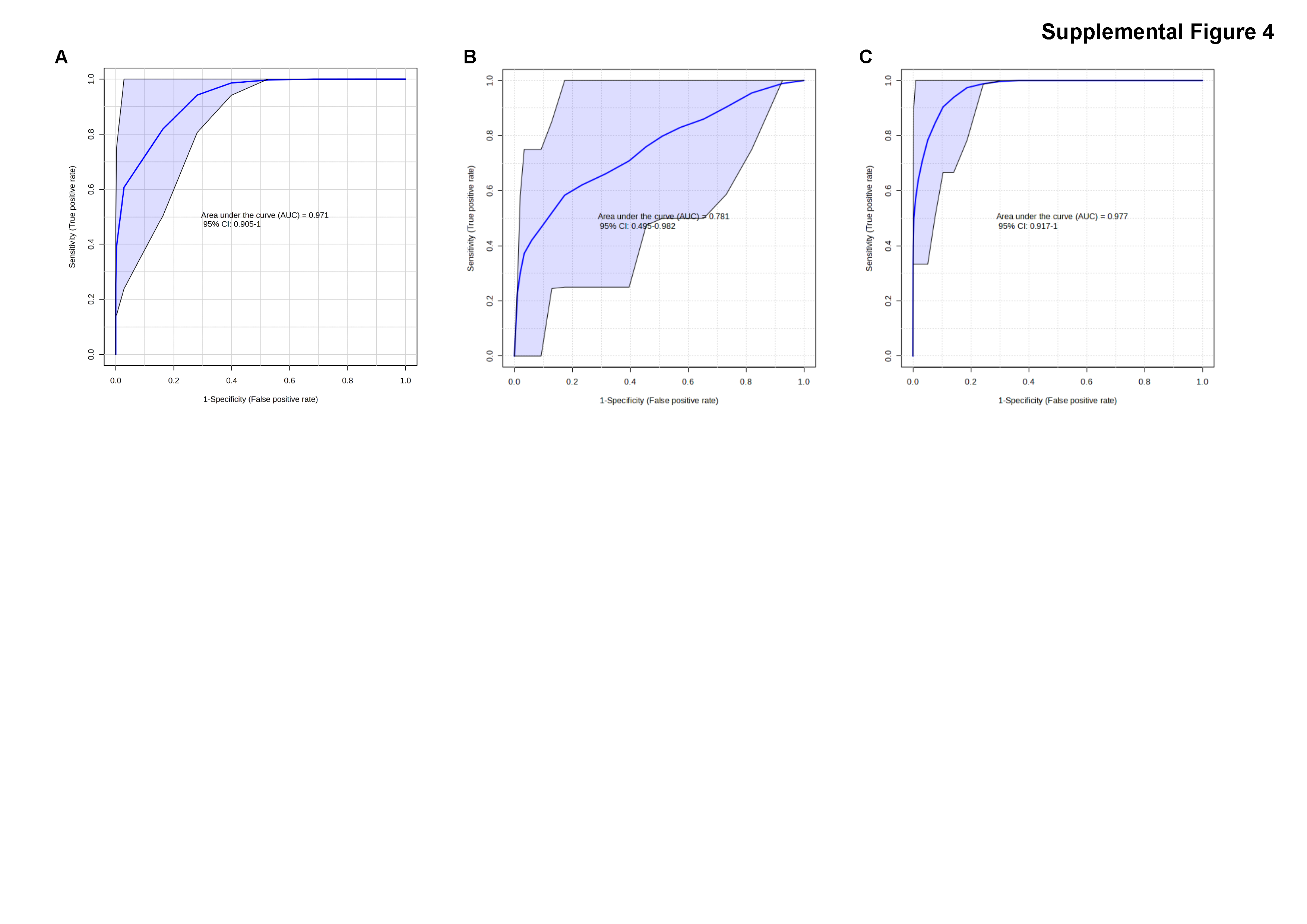

Supplement: Supplementary Figure 4 — Combined ROC of most relevant small molecule biomarkers. (A) Combined ROC of Oxalic acid and alpha-Tocopherol for the comparison between CA and COV/CA group; (B) Combined ROC of Arachidonic acid and alpha-Tocopherol for the comparison between COV and COV/CA patients; (C) Combined ROC of alpha-Tocopherol, 3-Indoleacetic acid, Arachidonic acid, Tryptamine and L-Tryptophan for the comparison between HD and COV patients. [file Image_4.tif]

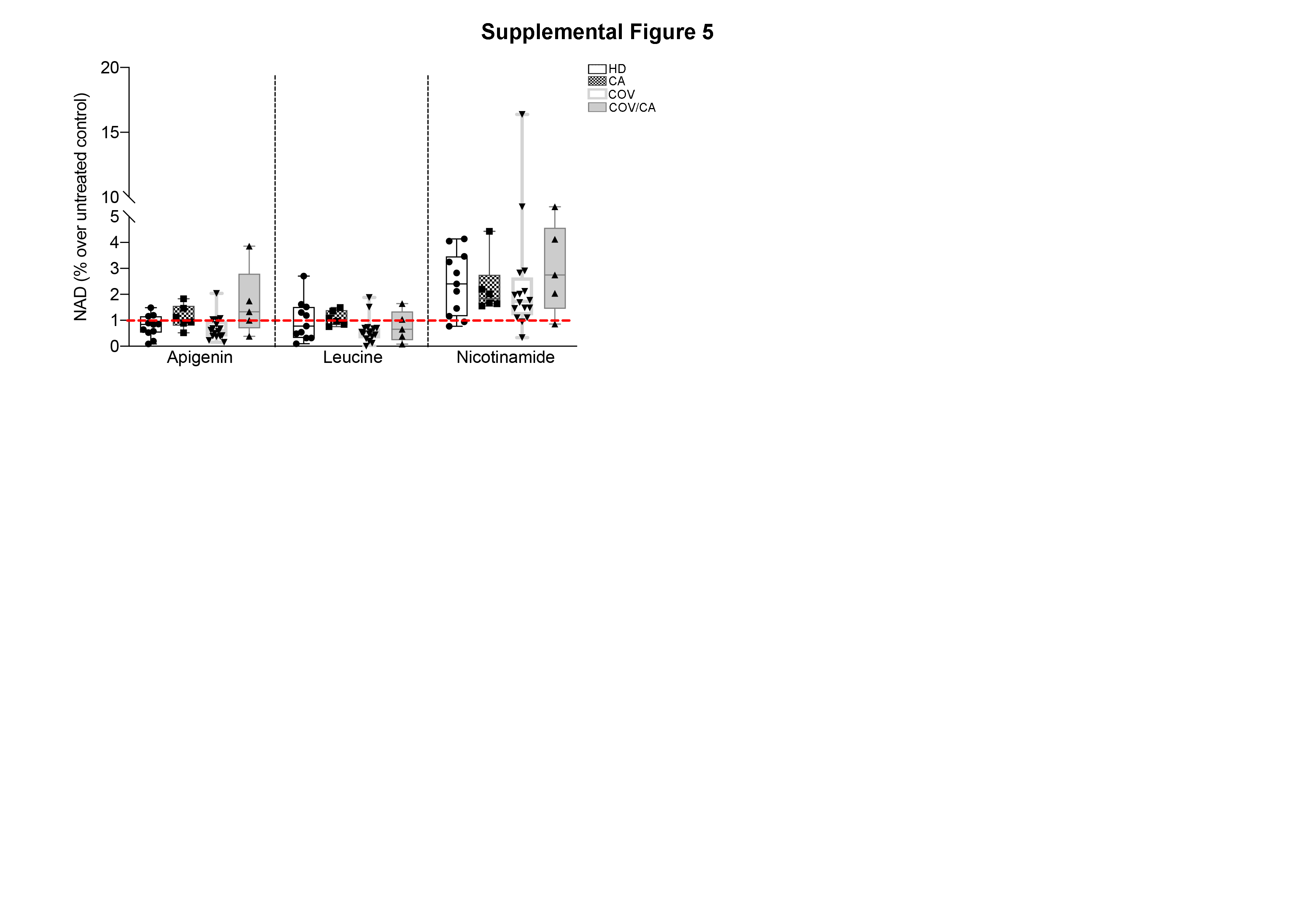

Supplement: Supplementary Figure 5 — Intracellular NAD+ level variations express as fold change over the relative control in HD, CA, COV and COV/CA patients. Isolated PBMCs were cultured in RPMI media or stimulated with 100 µM Apigenin or 500 µM Leucine or 800 µM Nicotinamide for 1 h before intracellular NAD+ quantification. HD (n = 11), CA (n = 6), COV (n = 16) and COV/CA (n = 5) patients. Statistical significance of differences between patient groups was calculated using Two-way ANOVA with Sidak’s multiple comparisons test or one-way ANOVA followed by Tukey’s multiple comparison test. *P < 0.05, **P < 0.01, ***P < 0.001, ****P < 0.0001 between selected relevant comparisons. [file Image_5.tif]

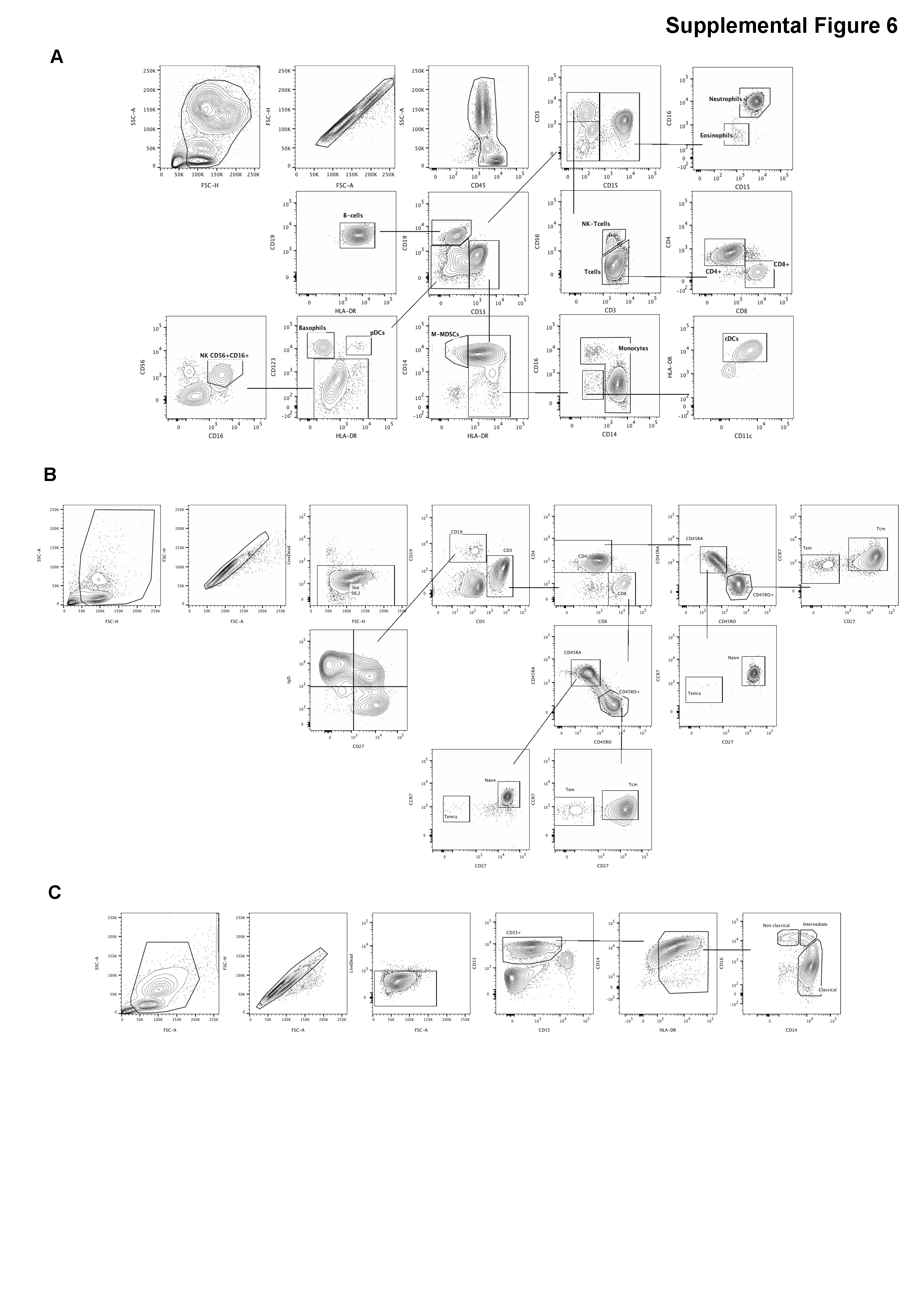

Supplement: Supplementary Figure 6 — (A) Representative flow cytometry gating strategy for the identification of major immune cell populations in blood. (B, C) Leucocyte gating strategy for phenotypic characterization of lymphocytes (B) and monocytes in PBMCs (C). [file Image_6.tif]
